# Mitochondrial dynamics dysfunction and neurodevelopmental disorders: From pathological mechanisms to clinical translation

**DOI:** 10.4103/NRR.NRR-D-24-01422

**Published:** 2025-06-19

**Authors:** Ziqi Yang, Yiran Luo, Zaiqi Yang, Zheng Liu, Meihua Li, Xiao Wu, Like Chen, Wenqiang Xin

**Affiliations:** 1 Queen Mary School, Jiangxi Medical College, Nanchang University, Nanchang, Jiangxi Province, China; 2 Department of Neurosurgery, First Affiliated Hospital of Nanchang University, Nanchang, Jiangxi Province, China

**Keywords:** autophagic clearance, autism spectrum disorders, cellular homeostasis, fusion and fission, mitochondrial dynamics, mitophagy, neural regeneration, neuronal energy metabolism, neurodevelopmental disorders, oxidative stress

## Abstract

Mitochondrial dysfunction has emerged as a critical factor in the etiology of various neurodevelopmental disorders, including autism spectrum disorders, attention-deficit/hyperactivity disorder, and Rett syndrome. Although these conditions differ in clinical presentation, they share fundamental pathological features that may stem from abnormal mitochondrial dynamics and impaired autophagic clearance, which contribute to redox imbalance and oxidative stress in neurons. This review aimed to elucidate the relationship between mitochondrial dynamics dysfunction and neurodevelopmental disorders. Mitochondria are highly dynamic organelles that undergo continuous fusion and fission to meet the substantial energy demands of neural cells. Dysregulation of these processes, as observed in certain neurodevelopmental disorders, causes accumulation of damaged mitochondria, exacerbating oxidative damage and impairing neuronal function. The phosphatase and tensin homolog-induced putative kinase 1/E3 ubiquitin-protein ligase pathway is crucial for mitophagy, the process of selectively removing malfunctioning mitochondria. Mutations in genes encoding mitochondrial fusion proteins have been identified in autism spectrum disorders, linking disruptions in the fusion-fission equilibrium to neurodevelopmental impairments. Additionally, animal models of Rett syndrome have shown pronounced defects in mitophagy, reinforcing the notion that mitochondrial quality control is indispensable for neuronal health. Clinical studies have highlighted the importance of mitochondrial disturbances in neurodevelopmental disorders. In autism spectrum disorders, elevated oxidative stress markers and mitochondrial DNA deletions indicate compromised mitochondrial function. Attention-deficit/hyperactivity disorder has also been associated with cognitive deficits linked to mitochondrial dysfunction and oxidative stress. Moreover, induced pluripotent stem cell models derived from patients with Rett syndrome have shown impaired mitochondrial dynamics and heightened vulnerability to oxidative injury, suggesting the role of defective mitochondrial homeostasis in these disorders. From a translational standpoint, multiple therapeutic approaches targeting mitochondrial pathways show promise. Interventions aimed at preserving normal fusion-fission cycles or enhancing mitophagy can reduce oxidative damage by limiting the accumulation of defective mitochondria. Pharmacological modulation of mitochondrial permeability and upregulation of peroxisome proliferator-activated receptor gamma coactivator 1-alpha, an essential regulator of mitochondrial biogenesis, may also ameliorate cellular energy deficits. Identifying early biomarkers of mitochondrial impairment is crucial for precision medicine, since it can help clinicians tailor interventions to individual patient profiles and improve prognoses. Furthermore, integrating mitochondria-focused strategies with established therapies, such as antioxidants or behavioral interventions, may enhance treatment efficacy and yield better clinical outcomes. Leveraging these pathways could open avenues for regenerative strategies, given the influence of mitochondria on neuronal repair and plasticity. In conclusion, this review indicates mitochondrial homeostasis as a unifying therapeutic axis within neurodevelopmental pathophysiology. Disruptions in mitochondrial dynamics and autophagic clearance converge on oxidative stress, and researchers should prioritize validating these interventions in clinical settings to advance precision medicine and enhance outcomes for individuals affected by neurodevelopmental disorders.

## Introduction

Neurodevelopmental disorders (NDDs) are a group of highly heritable disorders that exhibit a wide range of neurological and psychiatric symptoms from the onset of development (Thapar et al., 2017; Tanaka and Chung, 2025; Yin et al., 2025). The clinical features of NDDs mainly include autism, epilepsy, cerebral palsy, intellectual disability, deficits in social communication and interaction, and restricted and repetitive patterns of behavior, interests, and activities (Sahin and Sur, 2015). According to the Diagnostic and Statistical Manual of Mental Disorders (DSM-V), NDDs include autism spectrum disorder (ASD), intellectual disability, attention deficit hyperactivity disorder (ADHD) (Martinez and Peplow, 2024; Yan et al., 2024), specific learning disabilities, communication disorders, and movement disorders (Wakefield, 2016). NDDs, which are more common in males and usually occur in childhood before puberty, account for approximately 25% of chronic pediatric disorders and are the third-most common form of childhood disability after visual and hearing impairments (Rutter et al., 2003; Sabariego-Navarro et al., 2022; Sokolowski and Levine, 2023). NDDs have an early onset and long duration of illness and place a substantial economic burden on the families of affected children (Leigh and Du, 2015). The clinical course of NDDs differs from those of other neurological disorders in that symptoms show remission or relapse after puberty, instead presenting a stable learning and communication disorder. While NDDs are highly heterogeneous in terms of their clinical features, etiology, and treatment, the high degree of overlap between the disorders provides a supportive rationale for studying them together.

Mitochondria are semiautonomous organelles with multiple functions and are involved in a wide range of cellular processes that require the production of large amounts of adenosine triphosphate (ATP) through oxidative phosphorylation to supply energy requirements, especially in cells with high energy demands. The brain has been traditionally assumed to consume nearly 20% of the total body energy despite accounting for only 2% of the overall weight and has even higher energy requirements during development (Attwell and Laughlin, 2001). Mitochondria are double-membrane structures with an intermembrane space separating the outer and inner mitochondrial membranes. The inner mitochondrial membrane forms vesicular structures through invaginations of cristae. To adapt to energy demands, mitochondria undergo constant and highly dynamic changes, including fusion, fission, and movement (Bertholet et al., 2016). A variety of NDDs have been proposed to be attributable to disturbances in mitochondrial fission and/or fusion mechanisms. Thus, maintaining fission and fusion homeostasis is critical for health. Another important factor in maintaining mitochondrial health is mitochondrial autophagy, which is responsible for restoration or removal of damaged or dysfunctional mitochondria. Ubiquitin molecules promote the formation of autophagosomes around mitochondria by binding to proteins on the surface of damaged mitochondria. Subsequently, these autophagosomes are delivered to lysosomes for final degradation (Yoo and Jung, 2018; Onishi et al., 2021).

A growing body of evidence suggests that dysregulated mitochondrial autophagy is closely associated with NDDs. In addition to their roles in determining the location of mitochondrial morphology and mitochondrial autophagy-mediated clearance, mitochondria are involved in multiple processes such as bioenergetic metabolism, inward flow of Ca^2+^, regulation of permeability translocation pores, and biosynthesis (Rangaraju et al., 2019). Mitochondria play key roles in various stages of neural development, and their dynamic distribution and function directly affect the formation, maturation, and maintenance of nerve cells (Farahani, 2024; Huang et al., 2024; Yan et al., 2024). In the proliferation stage of neural stem cells, mitochondria are distributed in the cytoplasm and concentrated in the active areas of division, and they generate ATP through oxidative phosphorylation to support DNA replication and cell division (Coelho et al., 2022; Na et al., 2023; Rimbert et al., 2023). Consequently, insufficient ATP results in decreased proliferation rate and premature depletion of neural stem cell pools (Saha et al., 2024; Wang et al., 2024a; Lin et al., 2025; Pervaiz et al., 2025). The balance between glycolysis and oxidative phosphorylation affects stem cell fate, and a hypoxic environment may maintain stem cell pluripotency through glycolysis (Ren et al., 2024; Moiz et al., 2025). A variety of metabolic intermediates are involved in histone acetylation and affect gene expression (Sun et al., 2025; Tang et al., 2025). Metabolic abnormalities may lead to premature differentiation or apoptosis of neural stem cells, resulting in a variety of adverse consequences (Qiu et al., 2023; Lisowski et al., 2024; Tulva et al., 2025). During nerve cell differentiation, mitochondria aggregate around the cell body to support protein synthesis (Vujovic et al., 2024; Norouzi Esfahani et al., 2025). Simultaneously, mitochondrial oxidative phosphorylation is enhanced to promote the expression of nerve cell-specific genes (Ruan et al., 2023; Matrella et al., 2024). Blockage of nerve cell differentiation can lead to a decrease in the number of nerve cells and abnormal glial cell proliferation (Wang et al., 2025a, b). Moderate levels of reactive oxygen species (ROS) can promote nerve cell differentiation (Mitra et al., 2020), but excessive ROS levels can induce oxidative damage, cause DNA damage, and increase the risk of cell death (Silva et al., 2024; Chen et al., 2025b; Zhang et al., 2025a). In addition, mitochondria can also absorb cytoplasmic calcium and regulate calcium-dependent differentiation signals, thereby promoting nerve cell differentiation (Keilhoff et al., 2021; Sun et al., 2022; Whitehead et al., 2025). When nerve cells migrate, mitochondria migrate to the front of migration, near the center of microtubule tissue, providing ATP for actin and microtubules for energy supply (Bakaeva et al., 2024; Ma et al., 2024; Ribeuz et al., 2024). Delayed migration or mislocalization caused by mitochondrial dysfunction often results in abnormal cortical stratification (Khadimallah et al., 2022; Uspalenko et al., 2023). In addition, mitochondria also regulate local calcium concentration, which affects the activity of cell polar proteins. Calcium imbalance may lead to stagnation of migration (Li et al., 2024; Nadeem et al., 2024). Other studies have shown that mitochondria can respond to chemical chemokines to regulate migration pathways (Chen et al., 2024; Daga et al., 2024). During the growth and guidance of axons and dendrites, mitochondria are transported along microtubules to growth cones, enriched at branching points, supporting cytoskeleton extension and membrane transportation. Obstacles in this process may lead to misguided axons (Ibrahim et al., 2025; Marzetti et al., 2025). During this process, mitochondria generate nicotinamide adenine dinucleotide (NADH) and ATP, maintain high-energy consumption processes such as vesicle transportation in the growth cone (Pan et al., 2025; Wang et al., 2025d), and also regulate the sensitivity of guide molecules through ROS, which affects the growth direction (Nakamura et al., 2023). Mitochondrial dysfunction can lead to reduced dendritic complexity (Mishra and Thakur, 2024; Ramírez et al., 2025). During synaptic formation and pruning, mitochondria aggregate at the presynaptic terminals and dendritic spine bases, providing ATP for synaptogenesis and the synthesis of neurotransmitters such as glutamate and synaptic proteins (Islam et al., 2024; Tripathi and Ben-Shachar, 2024). Mitochondria regulate the activity of N-methyl-D-aspartate receptors through calcium uptake, affect long-term potentiation/long-term depression, and can also regulate construction, release cytochrome c and other pro-apoptotic factors, and participate in redundant synaptic clearance (Devine et al., 2022; Wang et al., 2024d; Samanta et al., 2025). Mitochondrial disorders in this process can lead to abnormalities in synaptic density, such as hyperpruning in schizophrenia, or dopaminergic synaptic failure in neurotransmitter release disorders such as in Parkinson’s disease (Hwang et al., 2024; Kambali et al., 2024; Sarnyai and Ben-Shachar, 2024; Abhilash et al., 2025). During the process of synaptic plasticity and maintenance of mature nerve cells, mitochondria are dynamically distributed near synapses and gather rapidly in response to changes in activity (Gupta et al., 2025; Pannoni et al., 2025). Mitochondria continuously supply energy for high-frequency discharge and synaptic vesicle circulation, and can also mediate antioxidant defense, clear ROS through catalase and superoxide dismutase, and protect synaptic structure (Liu et al., 2024; Almikhlafi et al., 2025; Musyaju et al., 2025). Thus, defects in mitochondrial function during these processes may cause a decline in synaptic function, leading to cognitive impairment and oxidative damage that accelerates neurodegeneration (Rezaee et al., 2025; Silvia et al., 2025a, b; Zhang et al., 2025b).

The common mechanisms of mitochondrial defects leading to neurological dysfunction can be mainly summarized as follows: First, in an energy crisis, ATP deficiency will affect the high-energy consumption process of ion pump and vesicle transportation (Mandal et al., 2025; Yousefian-Jazi et al., 2025). Second, dysregulation of calcium homeostasis can result in a decrease in the mitochondrial calcium-buffering capacity, leading to excitotoxicity or abnormal signaling (Cai et al., 2025; Chemla et al., 2025). ROS accumulation is another important reason. Oxidative damage to lipids, proteins, and DNA can often trigger apoptosis or inflammation (Li et al., 2025a, b; Spina et al., 2025). Moreover, transportation disorders and dynein or microtubule abnormalities can cause imbalances in the distribution of mitochondria (Chen et al., 2021; Vaillant-Beuchot et al., 2024; Guerra San Juan et al., 2025). Finally, epigenetic alterations such as metabolite deficiencies affect histone/DNA modifications, which in turn interfere with gene expression (Chen et al., 2023; Chodari et al., 2024). Mitochondria are involved in the entire course of neurodevelopment through spatiotemporal-specific energy metabolism, signal regulation, and structural support. Thus, mitochondrial defects can disrupt key developmental nodes and lead to widespread neurological dysfunction, highlighting the central role of mitochondria in nervous system health. Future studies should further analyze the molecular targets of mitochondria in specific developmental stages to provide a basis for intervention. NDDs often lead to impaired cognitive function, affecting cognitive and learning abilities (Hilz et al., 2025; Moll and Krishnan, 2025). For example, people with intellectual disabilities develop deficits in intellectual and adaptive function before 18 years of age, which seriously affect their academic performance and knowledge acquisition (Diril et al., 2025). In addition, patients with ADHD often experience difficulty in learning due to inattention, impulsivity, and hyperactivity, and 60%–80% of patients experience symptoms that persist into adulthood (Wang et al., 2025c). Lack of social skills is one of the common manifestations of NDDs. For example, people with ASD show significant impairments in social interaction and communication, limiting their ability to establish and maintain interpersonal relationships. This social impairment affects peer relationships in children and adolescents, and may also persist into adulthood, causing the patients to face significant challenges in social integration (Jain et al., 2025; Zhou et al., 2025). In addition, people with NDDs may face more mental health problems during adolescence. Research shows that adolescents with NDDs such as ADHD, ASD, and obsessive-compulsive disorder are more susceptible to emotion disorders and behavioral problems during adolescence (Hao et al., 2025). For example, women with NDD in late adolescence are more likely to exhibit internalizing behaviors, such as anxiety and depression, while men may exhibit externalizing behaviors, such as aggression, in early adolescence (Kohls et al., 2025; Zaguri-Vittenberg et al., 2025). In addition, people with obsessive-compulsive disorder experience tremendous psychological stress due to obsessive-compulsive thinking and behavior after onset in childhood and adolescence (Pinciotti et al., 2024; Towner et al., 2024). In addition to their current effects on the lives of children and adolescents, NDDs can also have long-term effects on the mental health and quality of life of these individuals in adulthood. Without timely intervention, some patients may develop lifelong disabilities. For example, childhood-onset epileptic encephalopathy can lead to mental retardation and brain atrophy, which can substantially affect a patient’s long-term prognosis (Specchio and Auvin, 2025; Zubal et al., 2025). The treatment and management of NDDs requires long-term investment, imposing economic and psychological burdens on families. Early diagnosis and intervention are essential to improve prognosis. However, the pathogenesis of NDDs is not yet clear, and drug treatments have been shown to have limited effects (Długosz et al., 2025; Puljko et al., 2025).

This review synthesizes the clinical, genetic, and molecular evidence, collects the data obtained using multi-omics analysis methods such as genomics, transcriptomics and proteomics, summarizes a variety of model systems, systematically explores the dynamic dysfunction of mitochondria and the role of mitochondrial autophagy in various NDDs, and reveals the key molecular mechanisms in these processes. Dynamic changes of mitochondria and mitochondrial autophagy are essential for maintaining energy metabolism of nerve cells and the stability of the intracellular environment. In nerve injury or disease states, mitochondrial dysfunction can lead to damage and death of nerve cells, and restoring mitochondrial function may help promote nerve regeneration (Wang et al., 2024b; Chauhan et al., 2025). In addition, this paper further reviews the pathogenesis and potential intervention targets of NDDs from the perspective of mitochondria, and summarizes a variety of therapeutic strategies based on mitochondrial function regulation, such as regulation of mitochondrial permeability and fusion kinetics and activating peroxisome proliferator-activated receptor gamma coactivator 1-alpha (PGC-1α). These strategies will provide new directions for the treatment of NDDs and yield potential clues for neural regeneration research.

In addition, this paper further reviews the pathogenesis of NDD and highlights potential intervention targets from the mitochondrial perspective, summarizing therapeutic strategies centered on regulating mitochondrial function. These strategies can provide new directions for treating NDDs and offer potential insights for neural regeneration research. Looking ahead, the field must strengthen the integration of basic research and clinical applications, encourage multidisciplinary collaboration, identify specific biomarkers, develop targeted therapies, and improve patient prognoses and quality of life at an early stage. Therefore, the primary purpose of this review is to present a comprehensive overview of the contributions of mitochondrial dynamics and mitophagy to NDD pathogenesis, describe relevant therapeutic strategies, and highlight avenues for future investigation.

## Search Strategy

A computer-based online search of relevant databases was performed to retrieve articles exploring the relationship between mitochondrial dysfunction and neurodevelopmental disorders (NDDs) that were published up to February 20, 2025. To maximize search specificity and sensitivity, a combination of the following text words (MeSH terms) was used: “mitochondrial dysfunction,” “mitochondrial dynamics,” “mitochondrial fusion and fission,” “mitochondrial autophagy,” “mitochondrial permeability,” “mitochondrial bioenergetics,” “mitochondrial calcium influx,” “mitochondrial oxidative phosphorylation,” “mitochondrial DNA mutations,” “mitochondrial transport,” “neurodevelopmental disorders,” “autism spectrum disorders (ASD),” “attention-deficit/hyperactivity disorder (ADHD),” “Rett syndrome,” “Down syndrome,” “Fragile X syndrome,” “mitochondrial quality control,” “PGC-1α activation,” “PINK1/Parkin pathway,” “neuronal energy metabolism,” “neuroinflammation,” “epigenetic modification,” “neural regeneration,” “therapeutic targets,” “mitochondrial ROS production,” “mitochondrial biogenesis,” “mitochondrial respiratory chain,” “mitochondrial dysfunction mechanisms,” “neurodevelopmental pathology,” “mitochondrial dysfunction treatment,” “mitochondrial dysfunction animal models,” “mitochondrial dysfunction clinical evidence,” “mitochondrial dysfunction biomarkers,” and “mitochondrial dysfunction personalized therapy. ” The results were further screened by title and abstract, and only those studies exploring the mechanisms, clinical evidence, and potential therapeutic strategies related to mitochondrial dysfunction in NDDs were included to summarize the contributions of both cellular and molecular components to the pathogenesis of these disorders. Most of the selected studies (72% of all references) were published in or after 2018. Articles focusing solely on other aspects of NDDs without addressing mitochondrial dysfunction or those unrelated to neurodevelopmental pathology were excluded.

## Mitochondrial Fusion/Splitting and Neurodevelopmental Disorders

The mechanisms of neurodevelopmental disorders triggered by different mitochondrial defects are detailed in **Table 1**.

**Table 1:** Mechanisms of neurodevelopmental disorders triggered by different mitochondrial defects

Type	Neurodevelopmental disorder	Mitochondrial defect mechanism
Mitochondrial fission	FOXP1 syndrome	Haploinsufficiency of the *FOXP1* gene leading to striatal dysfunction
	Leukodystrophy	Deficiency of the enzyme DEGS1 localized in the mitochondria-associated ER
Mitochondrial fusion	Bosch-Boonstra-Schaaf optic atrophy syndrome	Mutation in the *NR2F1* gene
	Leukodystrophy	Mutation in the ceramide synthase DEGS1 localized in the ER
	FOXP1 syndrome	PGC -1α stimulates Mfn1 in response to increased mitochondrial outer membrane fusion; dysregulation triggers mitochondrial structural disruption and energy transport interruption
Mitochondrial transport	Bosch-Boonstra-Schaaf optic atrophy syndrome	Mutation in the *NR2F1* gene
	Schizophrenia	Neurons exhibit altered dendritic morphology and/or maintenance
	Schizophrenia 1	Interaction between TRAK1 and Miro1, and involvement in the mitochondrial calcium uniporter complex regulating mitochondrial matrix calcium levels
	Schizophrenia and bipolar disorder	Expression of DISC1 in multiple subcellular regions
Mitochondrial autophagy	Familial autosomal recessive parkinsonism	Mutations in PINK1 and PARK2
	ADHD	Copy number variations in PARK2 with duplications and deletions
	Congenital microcephaly, brain atrophy, and growth retardation	Biallelic variants in CLEC16A
	Early-onset progressive spastic ataxia	Biallelic pathogenic variants in VPS13D
	BPAN, RLS, ID, DEE, EOEE and west syndrome	Deficiency of the WDR45 gene
	Epilepsy	Deficiency of *Il1rl1* encoding ST33
	FXS	X-linked mutation or deletion in the *Fmr1* gene
	ADHD and moderate ID	p. E411D mutation
	West syndrome	p. V622G mutation
	Patients with epilepsy and mild ID	p. A272V mutation
	Koolen-de Vries syndrome	Variants in the *KANSL1* gene
Mitochondrial DNA mutations and encoding abnormalities	Cognitive and behavioral deficits	Mice with inherited mitochondrial DNA mutations exhibit abnormal brain development and increased susceptibility
	Bosch-Boonstra-Schaaf optic atrophy syndrome	Variants in the *NR2F1* gene
	Dubowitz-like syndrome	Mutations in the *NSUN2* gene
Reduced activity of key mitochondrial bioenergetic metabolic enzymes	Early-onset and slowly progressive deep sensory loss and sensory ataxia	Variants in COX20
	Symptoms similar to ASD	Inherited mitochondrial DNA mutations with non-synonymous mutations affecting oxidative phosphorylation Complex I
	Epileptic seizures and motor deficits	Mutations in *FARS2* gene
	Hereditary intellectual disability	Deficiency in *btb11*
	RTT	Mutations in methyl-CpG binding protein 2 (MECP2) on the X chromosome
Mitochondrial Ca^2+^ influx and mitochondrial permeability	ASD	Variants in the *LC25A12* gene

This table outlines the diverse mitochondrial defects associated with various NDDs, categorizing them by type of mitochondrial dysfunction. The table covers defects in mitochondrial fission, fusion, transport, autophagy, mitochondrial DNA mutations, bioenergetic enzyme deficiencies, and calcium influx regulation. ADHD: Attention-deficit/hyperactivity disorder; ASD: autism spectrum disorder; BPAN: beta-propeller protein-associated neurodegeneration; BTB11: BTB domain-containing protein 11; CLEC16A: C-type lectin domain-containing protein 16A; COX20: cytochrome c oxidase assembly protein 20; DEGS1: delta-5-desaturase 1; DISC1: disrupted in schizophrenia 1; DEE: developmental and epileptic encephalopathies; EOEE: early-onset epilepsy encephalopathy; FARS2: phenylalanyl-tRNA synthetase 2, mitochondrial; FOXP1: forkhead box protein P1; FXS: fragile X syndrome; ID: intellectual disability; Il1rl1: interleukin 1 receptor-like 1; KANSL1: KAT8-associated factor 1; LC25A12: solute carrier family 25 member 12; MECP2: Methyl-CpG-binding protein 2; Mfn1: mitofusin 1; NR2F1: nuclear receptor subfamily 2 group F member 1; NSUN2: Sun RNA methyltransferase family member 2; OXPHOS: oxidative phosphorylation; PARK2: Parkin: RBR E3 ubiquitin protein ligase; PINK1: PTEN-induced putative kinase 1; PGC-1α: peroxisome proliferator-activated receptor-gamma co-activator 1α; RTT: Rett syndrome; RLS: restless legs syndrome; ST33: suppressor of tumorigenicity 33; TRAK1: trak protein 1; VPS13D: Vacuolar protein sorting 13D; WDR45: WD repeat-containing protein 45.

### Mitochondrial fission and neurodevelopmental disorders

**Figure 1:**
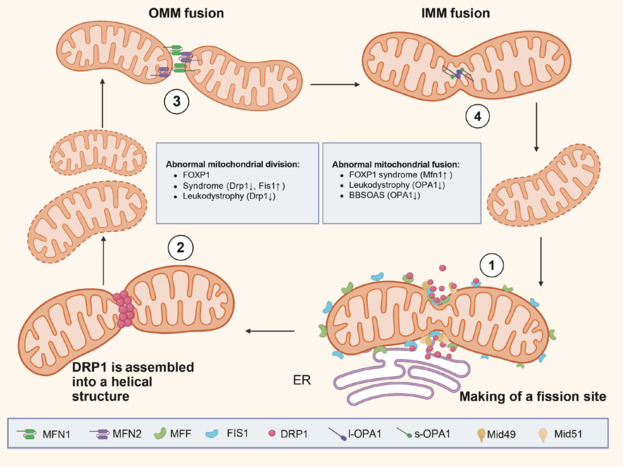
The process of mitochondrial fusion and division. At the ER contact site, the cytoplasmic GTP hydrolase dynamin-related protein 1 (Drp1) is recruited by four mitochondrial membrane proteins, Fis1, Mff, MiD49 and MiD51 (Thapar et al., 2017). Drp1, recruited to the mitochondrial surface, assembles into a helical structure that undergoes a process of wrapping and contraction, culminating in mitochondrial fission (Sahin and Sur, 2015). Mitochondria are double-membrane organelles, and fusion is divided into two steps:outer membrane fusion and inner membrane fusion. The proteases Mfn1 and Mfn2, located on the outer membrane of mitochondria, connect the outer membrane of two mitochondria to achieve outer membrane fusion. Endosomal fusion is mediated by Opa1, a member of the dynamin family. Opa1 in the endosomes is divided into the long type l-Opa1 and the short type s-Opa1, and the two proteins are interconnected to complete endosomal fusion. The mitochondrial matrix diffuses into the new mitochondria to complete the final fusion (Rutter et al., 2003). Created with BioRender.com. BBSOAS: Bardet-biedl syndrome–oculocerebrorenal syndrome of lowe–associated syndromes; DRP1: dynamin-related protein 1; ER: endoplasmic reticulum; FIS1: mitochondrial fission 1 protein; FOXO1: forkhead box protein O1; L-Opa1: long form of optic atrophy 1 protein; MFF: mitochondrial fission factor; Mfn1: mitofusin 1; Mfn2: mitofusin 2; Mid49: mitochondrial inner membrane protein Mid49; Mid51: mitochondrial inner membrane protein Mid51; OMM: outer mitochondrial membrane; IMM: inner mitochondrial membrane; S-Opa1: short form of optic atrophy 1 protein.

Cells can contain anywhere from tens to hundreds of mitochondria in varying numbers, each containing at least one mitochondrial DNA (mtDNA) genome. Since the generation of new mitochondria involves mtDNA replication, this process does not occur from scratch but must be derived from other mitochondria. Thus, mitochondrial fusion and division play important roles in restoring viability. In addition, individual mitochondria show a variety of morphologies, including spherical and tubular, and are of varying lengths. The determination of these morphologies is also controlled by the opposing processes of fusion and fission (Chan, 2020). The coordinated actions of mitochondrial fusion and fission represent a fundamental factor in maintaining mitochondrial shape, distribution, and size in response to changing conditions (**Figure 1**).

Mitochondrial fission involves the splitting into two smaller mitochondria mediated by multiple proteins. Dynamic protein-associated protein 1 (Drp1) is a guanosine triphosphate (GTP) hydrolase that plays a central role in mitochondrial fission (Pagliuso et al., 2018). Drp1 in the cytoplasm is recruited to the surface of the mitochondria and assembles into a helical structure, which undergoes a process of encapsulation and constriction, culminating in mitochondrial fission (Jimah and Hinshaw, 2019). Four major proteins, mitochondria fission protein 1 (Fis1), mitochondrial fission factor (Mff), mitochondrial dynamic protein 49 (MiD49), and MiD51, play recruitment roles in fission. Fis1, an anchoring protein, plays a linking role in yeast and is also expressed in mammals and is used to recruit Drp1 (Bui and Shaw, 2013). Mff, another anchoring protein, appears to play a simple role, with its expression showing a positive correlation with mitochondrial fission. When Mff is absent, it reduces the recruitment of Drp1 to the mitochondria, thereby impairing fission, whereas overexpression of Mff enhances mitochondrial fragmentation. In contrast to Mff, MiD operates in the opposite mode. Low-level MiD expression increases mitochondrial fission, and overexpression leads to mitochondrial lengthening. Both MiD and Mff independently anchor Drp1 to the mitochondrial outer membrane, but the role of the interactions between MiD and Mff in regulating fission appear to be more important. However, the exact mechanism underlying this coordinating effect is not clear (Palmer et al., 2013).

Drp1 plays a central role in the fission process, although dynamin-2 has been proposed to follow the action of Drp1 for further assessment of membrane rupture (Lee et al., 2016) in mammalian cells. Mitochondrial fission begins to occur when the GTPase Drp1 is recruited from the cytoplasm to the outer mitochondrial membrane via four known proteins: Fis1, Mff, MiD49, and MiD51. Fis1 serves as an interface protein between Drp1 and mitochondria and, although not as effective as the yeast Fis1, plays a role in coordinating mitochondrial fission and autophagy (Chan, 2020). Striatal dysfunction triggered by haploinsufficiency of the forkhead box protein P1 (*FOXP1*) gene underlies the neurodevelopmental disorder FOXP1 syndrome, which is characterized by functional deficits in locomotion, intelligence, and language. The reduction of phosphorylated Drp1 in FOXP1 is also indicative of the occurrence of reduced mitochondrial fission. Interestingly, however, an increase in Fis1 expression is associated with mitochondrial fragmentation (Misgeld and Schwarz, 2017). In mammalian cells, fission occurs preferentially in regions in contact with the endoplasmic reticulum (ER) due to ER-associated inverted formin 2-mediated actin polymerization (Friedman et al., 2011). Actin filaments accumulate between mitochondria and inverted formin 2-rich ER membranes at the site of contraction, driving initial mitochondrial contraction, which allows Drp1-driven secondary contraction. In addition, deficiency of delta-5-desaturase 1, a mitochondria-associated ER-localizing enzyme, induces cerebral white matter dystrophy (Korobova et al., 2013). Upon starvation, as cellular cAMP levels increase, activated protein kinase A phosphorylates pro-fission Drp 1, which is retained in the cytoplasm to undergo mitochondrial fusion, thus sparing it from autophagic degradation (Gomes et al., 2011). Neural stem cells in the adult mammalian hippocampus are constantly generating new functional neurons that promote cognitive processes and emotional regulation, a process that is associated with extensive changes in mitochondrial mass, distribution, and shape. Enhancing Drp1 activity has been shown to further accelerate exercise-induced neuronal maturation (Steib et al., 2014).

### Mitochondrial fusion and neurodevelopmental disorders

The dynamic processes of mitochondrial fission and fusion are essential for maintaining mitochondrial homeostasis. When these processes are in equilibrium, the normal number of mitochondria and their rational morphological structure can be preserved. For instance, when fission is inhibited, mitochondria tend to increase interconnections and extend dramatically due to unopposed fusion. This phenomenon underscores the interdependence of fission and fusion mechanisms, which together form a feedback regulatory loop to ensure mitochondrial quality and function (Chen et al., 2003).

Mitochondria are double-membrane organelles, and fusion is preceded by fusion of the outer membrane and followed by fusion of the inner membrane. In most cases, outer- and inner-membrane fusion occur sequentially with a short interval. When the inner and outer membranes are fused, the mitochondrial matrix diffuses into the new mitochondria to complete the final fusion (Chan, 2020). Three GTP hydrolysates, mitofusin (Mfn)1, Mfn2, and optic nerve atrophy 1 (Opa1), support the fusion process. The mitochondrial proteases Mfn1 and Mfn2 are located on the outer mitochondrial membrane and are associated with outer membrane fusion. Mitochondrial inner membrane fusion is mediated by Opa1, a member of the dynamin family that is required for inner membrane fusion. Cells with aberrant Opa1 expression exhibit fragmentation characteristics despite completion of outer membrane fusion (Pernas and Scorrano, 2016). In addition, independent of facilitating mitochondrial fusion, Opa1 plays an important role in maintaining cristae structures. The cristae structure in mitochondria provides aggregation sites for respiratory chain super-complexes, and in the absence of Opa1, the cristae ultrastructure is severely disrupted, which, in turn, greatly reduces energy supply capacity (Cogliati et al., 2013).

Bosch-Boonstra-Schaaf optic atrophy syndrome, triggered by mutations in the nuclear receptor subfamily 2 group f member 1 (*NR2F1*) gene, is a rare neurodevelopmental disorder. Loss of NR2F1 function in adult mice induces downregulation of the levels of mitochondrial fusion-related proteins Mfn2 and Opa1, which triggers mitochondrial fragmentation in newborn neurons (Bonzano et al., 2023). Cerebral white matter dystrophy is an inherited white matter disorder induced by mutations in the ER sphingolipid desaturase delta-5-desaturase 1, which impacts motor function and cognition in early childhood (Köhler et al., 2018; Pant et al., 2019). Delta-5-desaturase 1 deficiency induces defective mitochondrial kinetics of over-fusion. Drp1 levels have been shown to be significantly reduced in Delta-5-desaturase 1-deficient patients, but Mfn2 levels were not significantly altered. Decreased Opa1, however, was associated with its shift to the shorter Opa1 isoform associated with increased fission (Planas-Serra et al., 2023). In addition, dysregulation of PGC-1α, which regulates mitochondrial biogenesis, in FOXP1 syndrome has also been shown to disrupt the balance between mitochondrial fusion and fission (Martin et al., 2014). PGC-1α stimulates Mfn1 in response to increased mitochondrial outer membrane fusion, and its dysregulation triggers mitochondrial structural disruption and disruption of energy transport; these changes induce FOXP1 syndrome, which is specifically characterized by mental retardation and language dysfunction (Wang et al., 2022b).

### Axonal transport and plasticity with neurodevelopmental disorders

**Figure 2:**
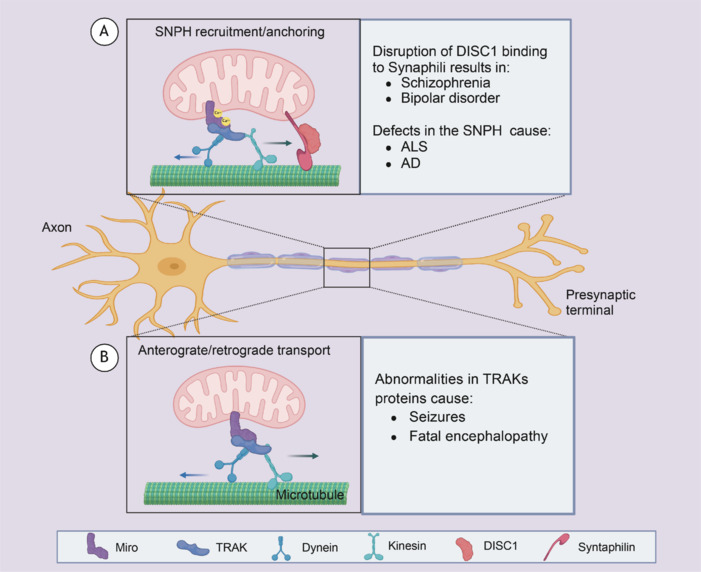
Mitochondrial axonal transport and plasticity abnormalities in neurodevelopmental disorders. In neurons, mitochondria travel long distances along microtubules driven by Kinesin-1 and the reverse dynein. Milton, also known as TRAK, acts as a linker between Miro, a protein anchored to the outer mitochondrial membrane, and the motor. (A) A high concentration of Ca^2+^ activates Miro recruitment to the anchoring protein Synaphili, thereby preventing mitochondrial motility. DISC1 can bind to Synaphili and relieve the restriction on mitochondrial movement. When DISC1 is abnormal, it leads to schizophrenia and bipolar disorder. (B) Abnormalities in connexin TRAK can induce seizures and fatal encephalopathy. Created with BioRender.com. AD: Alzheimer’s disease; ALS: amyotrophic lateral sclerosis; DISC1: disrupted in schizophrenia 1; Dynein: a type of motor protein; Kinesin: a type of motor protein; Miro: mitochondrial Rho GTPase 1; SNPH: synphilin-1; Syntaphilin: a protein that interacts with synphilin-1; TRAK: trak protein family.

In many cells, mitochondria are highly mobile and cross the cytoplasm by transport along the cytoskeleton. In newborn hippocampal neurons, mitochondria near synapses that act as a stable local energy supply play an active role in the maintenance of spine and synaptic morphogenesis and plasticity, as outlined in **Figure 2**. As mentioned in the study, mutations in the NR2F1 gene lead to a rare neurodevelopmental disorder, Bosch-Boonstra-Schaaf optic atrophy syndrome. In damaged neurons, depletion of NR2F1 expression, which is associated with the number of dendritic spines, is significantly reduced (Bonzano et al., 2023). Neurite formation is important for the formation of axons and dendrites in early neuronal development, and studies (Fung et al., 2011; Xia et al., 2024) have observed reduced neurite growth in neurons from patients with schizophrenia affecting neurodevelopment. Impaired neural network connectivity due to altered dendritic morphology growth and/or maintenance was observed in FOXP1 striatal neurons as a major cause of social and cognitive deficits, which is similar to the pathogenesis of ASD (Wang et al., 2022a).

Mitochondria move over long distances along microtubules via kinesin-1, a forward molecular motor of the kinesin family (also known as kinesin heavy chain), and dynein, a reverse molecular motor of the dynamin family (also known as the dynatin complex). Mitochondrial rho GTPase is a protein anchored to the outer membrane of the mitochondrion, and Milton acts as an adaptor between mitochondrial rho GTPase and motor function (Kang et al., 2008; Schwarz, 2013). Translocation driver proteins (TRAKs) support mitochondrial translocation in neurons. Relevant studies have reported that pathogenic TRAK1 variants cause disturbances in mitochondrial movement and distribution, leading to aberrant cellular respiration and ultimately causing fatal encephalopathies (Barel et al., 2017; Wu et al., 2021). TRAK1 interacts with mitochondrial rho GTPase 1 and acts concomitantly on the mitochondrial calcium uniporter complex that regulates mitochondrial mechanistic calcium ion concentrations and the influx of calcium into the mitochondrial matrix. However, this event is disrupted by schizophrenia 1 (DISC1) (Niescier et al., 2013). DISC1 is expressed in several subcellular compartments, including mitochondria, and is a risk factor for several neurological disorders such as schizophrenia and bipolar disorder (Ogawa et al., 2014).

Mitochondrial transport is critical because of the need for high energy during neurotransmission and the buffering of calcium ions along axons to synapses. The TRAK family of articulatory proteins (TRAK1 and TRAK2) that connect mitochondria to microtubule-based motors are required for axonal and dendritic mitochondrial motility (van Spronsen et al., 2013). Cells with defective TRAKs exhibit an irregular distribution of mitochondria and altered motility of paracrine and retrograde transport, and pathogenic variants leading to aberrant splicing and low-level expression of genes are associated with severe neurodevelopmental delays, epileptic seizures, and fatal encephalopathies (Barel et al., 2017).

The proper distribution of mitochondria within axons and at synapses is essential for neuronal function. The mitochondrial anchoring protein Synaphili is recruited to the outer mitochondrial membrane, connecting it to microtubules and preventing them from moving, a process that is a mandatory mitochondrial localization step. Synphilin-1 (SNPH)-mediated regulation of mitochondrial transport and localization is essential for axon outgrowth during neuronal development and for the maintenance of the ATP levels required for synaptic activity (Wu et al., 2023). The percentage of mobile axonal mitochondria is significantly higher in *SNPH* gene-deficient mice (Kang et al., 2008). Most of the current studies on the effects of SNPH deletions or variants on mitochondrial mobility have focused on neurodegenerative diseases (Zhu and Sheng, 2011; Han et al., 2020), such as amyotrophic lateral sclerosis-like disorders and Alzheimer’s disease, which lead to the accumulation of axons in damaged mitochondria, mainly through a prolonged stress response, and the selective release of SNPH proteins facilitates the stimulation of mitochondrial transport to the somatic cells (Lin et al., 2017). However, SNPH studies in NDDs are lacking.

## Mitochondrial Autophagy and Neurodevelopmental Disorders

In addition to mitochondrial dynamics, which include the processes of fusion, fission, and transport, selective degradation of dysfunctional and disordered mitochondria is essential as a control mechanism to ensure the quality of healthy mitochondria.

### The autophagy process involves the membrane-linked proteins PARL, PINK1, and Parkin

Mutations in PTEN-induced putative kinase 1 (PINK1) and PARK2, a E3 ubiquitin ligase also known as Parkin, have been identified to contribute to familial recessive Parkinson’s syndrome (Kitada et al., 1998). PINK1 is a protein kinase, and Parkin is an E3 ubiquitin ligase (Koyano et al., 2014). PINK1 phosphorylates ubiquitin on the mitochondrial outer membrane at serine residue 65, forming phosphorylated ubiquitin with high affinity for Parkin. This drives the recruitment of Parkin on the outer mitochondrial membrane and the subsequent conformational change, thereby catalyzing ubiquitin translocation and ubiquitin chain labeling and triggering autophagic clearance of damaged mitochondria (Koyano et al., 2014; Shiba-Fukushima et al., 2014; Okatsu et al., 2015; Nguyen et al., 2016). Labeled mitochondria use ubiquitin chains as molecular signals to recruit autophagy receptors. Five receptors are involved in PINK1/Parkin mitochondrial autophagy: optic nerve phosphatase, nuclear dot protein 52, P62, and Tax 1 binding protein 1 (Chan et al., 2011; Sarraf et al., 2013). Autophagy receptors undergo phosphorylation by binding to TANK-binding kinase 1 to drive their binding to members of the autophagy-associated protein 8 family (microtubule-associated protein 1 light chain 3 [LC3] and gamma-aminobutyric acid receptor-associated protein [GABARAP]), which play roles in late autophagosome extension and sealing (Wild et al., 2011; Itakura et al., 2012). After PINK1/Parkin activation, the autophagosome initiation kinase unc-51-like autophagy-activated kinase 1 and the vesicle-associated protein autophagy-associated protein 9a enable the recruitment of pre-formed barrier membranes to the mitochondrial surface to initiate autophagosome formation (Lazarou et al., 2015). Tubulin binding factor D 15 and tubulin binding cofactor D 17 are activated and then connected to the outer mitochondrial membrane protein Fisl, which is activated by the LC3/GABARAP interaction to achieve a regulatory role in autophagosome formation around mitochondria, with the Ras-related protein Rab-7 playing a fine-tuning role in this process. In FOXP1 syndrome, increased PINK1 and Parkin expression support autophagy of damaged mitochondria, and the expression of the microtubule-associated protein LC3A, which promotes the assembly of autophagosomes, is significantly elevated (Wang et al., 2022a).

Patients with ADHD who carry PARK2 copy number variation duplications and deletions have been diagnosed with mitochondrial dysfunction and abnormal energy metabolism, showing lower cellular ATP levels and reduced oxygen consumption rates. In addition, energy impairments in these patients may be related to the role of PARK2 dysregulation in affecting mitochondrial dynamics (Palladino et al., 2020). The E3-ubiquitin ligase C-type lectin domain-containing protein 16A (CLEC16A) is a deleterious variant associated with severe NDDs. Four pre-existing individuals in families with a double-allele CLEC16A variant showed features of congenital microcephaly, brain atrophy, and growth retardation. In a study of zebrafish embryonic brain development, deletion of CLEC16a was found to induce accumulation of damaged mitochondria and dysregulation of mitochondrial autophagy through inhibition of Parkin turnover and proteasomal degradation (Smits et al., 2023). Vacuolar protein sorting 13D (VPS13D) is a ubiquitin-binding protein that plays an important role in regulating mitochondrial autophagy. Recent studies have shown that a double-allele pathogenic variant of VPS13D is the genetic cause of early-onset progressive spastic ataxia (Seong et al., 2018; Durand et al., 2022). VPS13d mutants inhibit the onset of fusion by increasing the expression of the regulator of mitochondrial fusion, Mfn2, and regulating the mitochondrial and ER contact points and its downstream fission factor, Drp1, thereby controlling mitochondrial size and triggering autophagic clearance (Wang and Zhang, 2018; Shen et al., 2021a). Mitochondrial autophagic clearance is impaired by the loss of VPS13D. VPS13D was found to affect the mitochondrial autophagy pathway by regulating autophagy-associated protein 8a and ubiquitin localization in the mitochondrial core autophagy machinery. Furthermore, loss of VPS13d causes an autophagy defect like that of PINK1 deletion and a different autophagy manifestation than the loss of Parkin (Shen et al., 2021b).

### Autophagy removes damaged mitochondria and prevents the increase in reactive oxygen species

**Figure 3:**
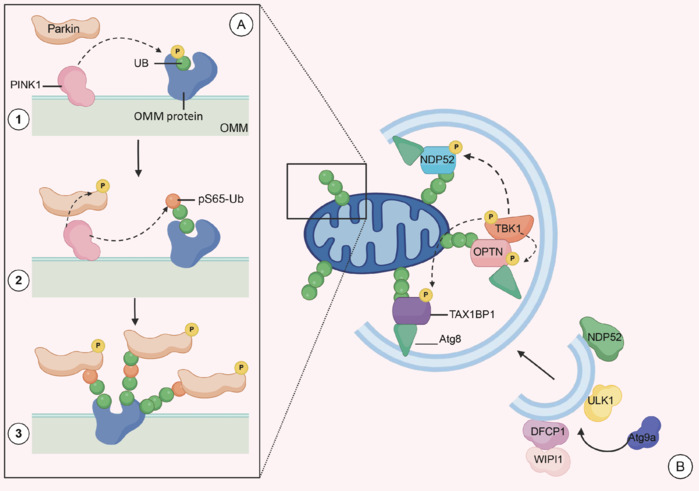
The process of mitochondrial autophagy. (A) PINK1/Parkin-mediated mitochondrial ubiquitination. Ubiquitin is linked to proteins. The outer mitochondrial membrane at the S65 site and PINK1 initiates recruitment of the ubiquitin ligase Parkin by phosphorylating (indicated by dashed arrows) ubiquitin (Ub) (Sabariego-Navarro et al., 2022). Parkin’s high affinity for s65-phosphorylated ubiquitin (pS65-Ub) drives a conformational change in its translocation to the outer mitochondrial membrane, from a closed structure to a stable open conformation, relieving activity inhibition (Sahin and Sur, 2015). Activated Parkin attaches ubiquitin to outer mitochondrial membrane proteins, providing more ubiquitin substrate for PINK1 phosphorylation. At the same time, the phosphorylated ubiquitin substrate activates the recruitment of more Parkin molecules. The recruited Parkin couples more ubiquitin to the outer mitochondrial membrane protein. This positive feedback loop promotes continued recruitment and amplification of signaling molecules (Battle, 2013). (B) A model of autophagosome formation during mitochondrial autophagy. The s65-phosphorylated ubiquitin chain (pS65-Ub) produced by PINK1 and Parkin binds to the receptors OPTN, NDP52, and TAX1BP1. The binding partner of OPTN, TBK1, phosphorylates Optineurin, NDP52, and TAX1BP1 by completing autophosphorylation and phosphorylating Optineurin, NDP52, and TAX1BP1 (the process of phosphorylation is indicated by dashed arrows). Phosphorylation of the three receptors promotes their pair binding to pS65-Ub. Subsequently, Optineurin and NDP52 recruit autophagy-initiating kinase 1 (ULK1) to initiate autophagosome formation on damaged mitochondria. Simultaneously, autophagy-associated protein 9a (Atg9a) and lipid kinase complex are recruited. Upon initiation, the production of phosphatidylinositol 3-phosphate during recruitment of lipid kinase complex further recruits WIPI1 and DFCP1. The initial formation of the isolation membrane prepares for the formation of autophagic vesicles. Autophagy receptors bind to microtubule-associated proteins to promote the extension of the mitochondrial barrier membrane. Eventually, damaged mitochondria are encapsulated within the autophagosomes and delivered to the lysosome for degradation. Created with BioRender.com. Atg8: Autophagy-related protein 8; Atg9a: autophagy-related protein 9a; DFCP1: double FYVE domain-containing protein 1; NDP52: nuclear dot protein 52; OMM: outer mitochondrial membrane; OPTN: optineurin; Parkin: an E3 ubiquitin-protein ligase; PINK1: PTEN-induced putative kinase 1; pS65-Ub: phosphorylated serine 65–ubiquitin; TAX1BP1: Tax1-binding protein 1; TBK1: TANK-binding kinase 1; UB: ubiquitin; ULK1: Unc-51-like autophagy-activating kinase 1; WIPI1: WD repeat-containing protein interacting with phosphoinositide 1.

Maintenance of neuronal mass and functional stability depends on proper mitochondrial autophagic scavenging (**Figure 3**).

Mitochondrial dysfunction increases ROS accumulation and leads to cell death. Increased expression of ROS has been observed in patients with neuronal injury. In patients with white matter encephalopathy induced by delta-5-desaturase 1 variants, ROS production is increased in fibroblasts. Based on the reduction of nicotinamide adenine dinucleotide phosphate, an electron donor required for glutathione reductase, which regulates glutathione production, the low reducing power in FOXP syndrome induces and thus increases the levels of ROS (Wang et al., 2022a). Complex I deficiency is the most common cause of NDDs in humans. It mainly affects mitochondrial function, causing excessive accumulation of ROS and thereby inducing neuroligin-mediated neurodevelopmental defects. Lutein reduces the generation of ROS by mutations in ubiquinone oxidoreductase Fe-S protein 1 or 4, which regulate oxidoreductase activity (Maglioni et al., 2022). The WD repeat-containing protein 45 (WDR45) gene is located on the X chromosome and targets mitochondria and is required for selective autophagy. WDR45 deficiency leads to mitochondrial damage and oxidative stress, and variations in the gene have been associated with six different neurodegenerative disorders, namely β- propeller protein-associated neurodegeneration, Rett-like syndrome, intellectual disability, developmental and epileptic encephalopathy, early-onset epileptic encephalopathy, and West syndrome (Cong et al., 2021). To adapt to the changing needs of the developing brain, microglia must undergo corresponding morphological and functional changes and remodeling. During this process, microglia exhibit features that promote mitochondrial activity and phagocytic activation in a protein kinase B (AKT)-dependent manner. Dysfunction of interleukin (IL)-33 and its receptor ST2 leads to impaired microglia development and impaired synaptic function. In addition, conditional deletion of *Il1rl1*, which encodes ST2, increases susceptibility to seizures (He et al., 2022). Fragile X syndrome is a genetic disorder characterized by a range of cognitive and behavioral deficits, including mild-to-moderate mental retardation. The pathogenic mechanism of this syndrome involves the silencing of the gene encoding the fragile X mental retardation protein, a translational regulator essential for neurodevelopment, due to an X-linked mutation in the *Fmr1* gene. Studies have shown altered mitochondrial respiratory capacity and high levels of ROS accumulation characterized by *Fmr1* deletion in studies of fragile X syndrome patients (Vandenberg et al., 2022; Gonzalez et al., 2025). Three novel neo-CACNA1C variants (p. E411D, p. V622G, and p. A272V) cause neurodevelopmental deficits. p. E411D variants are found in patients with ADHD and moderate intellectual disability. p. V622G variants are seen in patients with West syndrome, whereas p. A272V variants are found in patients with epilepsy and mild intellectual disability. All three variants show disturbed calcium currents and abnormal mitochondrial copy number and ATP production. These variants impair mitochondrial and lysosomal function and ultimately accelerate impaired mitochondrial autophagy (Kessi et al., 2023). Gilles de la Tourette syndrome is a complex multifactorial neurodevelopmental disorder characterized by dyskinesia and impaired vocalization. However, the underlying etiology of this disorder remains largely unknown, and the influence of multiple genes accompanied by environmental factors is now commonly recognized. Mitochondrial inner membrane peptidase subunit 2 is one of the susceptibility genes for Tourette syndrome, and a significant increase in mitochondrial oxidative stress levels has been reported in inner membrane peptidase subunit 2-deficient mouse and human cells. However, in comparison to controls, lower mitochondrial dysfunction showed weak differences (Bjerregaard et al., 2020).

In NDDs with abnormal mitochondrial autophagy, neurodegeneration is exacerbated not only by ROS accumulation but also by dysfunctional mitochondrial clearance through impaired function. Koolen-de Vries syndrome is a neurodevelopmental disorder caused by haploinsufficiency of the KAT8-associated factor 1 (KANSL1) gene. Variations in the KANSL1 gene induce dysfunction of autophagy. Thus, accumulation of damaged mitochondria in neuronal cells is the pathogenesis of Koolen-de Vries syndrome. Furthermore, in KANSL1-deficient patients, the significantly reduced antioxidant enzyme superoxide dismutase 1 stimulates subsequent oxidative stress-mediated accumulation of autophagosomes, which further prevents autophagosome scavenging and disrupts synaptic and neuronal networks (Li et al., 2022; Linda et al., 2022).

## Molecular Mechanisms of Mitochondrial Abnormalities and Neurodevelopmental Disorders

### Effects of mtDNA mutations and coding abnormalities on neurodevelopmental disorders

In animal studies, mice born with inherited mtDNA mutation-deficient mice exhibit abnormal brain development and are more prone to cognitive and behavioral deficits (Yardeni et al., 2021; Wang et al., 2022a). Mutations in mtDNA led to the impaired synthesis and loss of function of proteins associated with the oxidative phosphorylation system, which leads to ATP depletion and ROS overproduction, a process that can, in turn, induce further mtDNA mutations. Variations in the NR2F1 gene in Bosch-Boonstra-Schaaf optic atrophy syndrome not only affect mitochondrial dynamics but also directly target genes important for mitochondrial DNA transcription and translation. These targeted genes include a key metabolic nuclear gene required for respiration and a major activator of mitochondrial DNA replication, nuclear respiratory factor 1, as well as the nuclear gene regulating mitochondrial function, estrogen-related receptor alpha. These changes indirectly affect the mitochondrial DNA encoding mitochondrially encoded cytochrome c oxidase I (Bonzano et al., 2023). For normal expression of mitochondrial genes, post-expression modification is a key indispensable step. The mitochondrial genome encodes 22 tRNAs, and cytosine-5 methylation in post-transcriptional modification is one of the key regulatory steps in mitochondrial gene expression. Mammalian NOP2/Sun RNA methyltransferase family member 2 (NSUN2) is essential to produce m50C by several mammalian mitochondrial tRNAs, and NSUN2 variants have been associated with NDDs (Van Haute et al., 2019). In several other families with NSUN2 mutations, the Dubowitz-like syndrome, which presents with exhibits mental retardation, growth retardation, microcephaly features, and a mutation in a conserved residue (p. Gly679Arg) by a pure missense variant of NSUN2, causes spasticity, ataxic gait, and developmental delayed dysmorphism in the child (Khan et al., 2012; Martinez et al., 2012). Therefore, the maintenance of mitochondrial genetic stability and the normal function of transcription and translation is crucial to prevent the development of NDDs, but the current relevant studies are not sufficient to clarify these processes.

### Reduced activity of key enzymes in mitochondrial bioenergetic metabolism affects energy metabolism in neural development

The end product of glycolysis, pyruvate, enters the mitochondria and is subsequently converted to acetyl CoA and eventually enters the tricarboxylic acid (TCA) cycle. The reduced form of flavin adenine dinucleotide (FADH) and NADH produced in the TCA cycle then donate the reduction product electrons to the electron transport chain in the inner mitochondrial membrane. The electron transport chain consists of four oxidative phosphorylation complexes. The electrons shuttle to eventually form water in complex IV, also known as cytochrome c oxidase (COX). Coenzyme Q and cytochrome c are responsible for moving electrons between complexes. Reducing equivalents are used to transfer electrons through subsequent members of the electron transport chain, including complexes I–IV, to produce electrochemical gradients. This sequence of events concludes with the generation of ATP from the energy stored in the electrochemical gradient in the presence of complex V, which maintains the energy supply required for life survival (van der Bliek et al., 2017; Yan et al., 2019).

Schizophrenia is a severe complex syndrome originating from NDD. It presents with psychotic symptoms such as hallucinations and delusions with cognitive dysfunction. When comparing mitochondria in schizophrenia and control samples of NSC under reduced consumption levels of functioning, non-mitochondrial oxygen consumption, ATP production, ROS levels, and respiratory capacity levels were elevated (Zuccoli et al., 2023). The current study found that allogeneic healthy mitochondrial transplants can exert a therapeutic effect in schizophrenic rats, a finding that demonstrates the important role of mitochondria in neurodevelopment and provides new insights into the role of mitochondrial transplants in the treatment of NDDs such as schizophrenia (Ene et al., 2023). COX20 is an assembly chaperone protein involved in the mitochondrial oxidative phosphorylation complex IV. Patients with the COX20 variants are characterized by early onset and slowly progressive loss of deep sensation and sensory ataxia. With reduced COX20 protein levels, impaired mitochondrial complex IV assembly induces defective oxidative phosphorylation function, which leads to mitochondrial bioenergetic dysfunction (Dong et al., 2021). Mice with hereditary mtDNA mutations and carrying non-synonymous mutations affecting oxidative phosphorylation complex I have deficits in social interactions (inadequate social communication), repetitive behaviors, and restricted interests similar to the symptoms of ASD. In addition, mitochondrial respiratory function and ROS levels in the hippocampus are similarly affected (Yardeni et al., 2021; Wang et al., 2022b). Mutations in the gene encoding mitochondrial aminoacyl-tRNA synthetase have been associated with a variety of diseases. Phenylalany1l-tRNA synthetase 2, mitochondrial (dFARS2) is the *Drosophila* homolog of mitochondrial phenylpropanoid-tRNA synthetase. dFARS2 is required for the aminoacylation of mitochondrial tRNAs, for the stability of mitochondrial proteins, and for the assembly and enzymatic activity of the oxidative phosphorylation complex. Interestingly, seizure behavior and motor deficits have been induced by simulating FARS2 mutations in *Drosophila* (Fan et al., 2021). Zinc finger and BTB domain-containing protein 11 (Zbtb11) is a conserved transcription factor mutated in families with inherited intellectual disabilities and plays an important regulatory role in mitochondria. Zbtb11 plays a critical role in the activation of respiratory complex I. Genetic inactivation of Zbtb11 leads to impaired recruitment of nuclear respiratory factor 2 (NRF-2) to its target promoter, severe defects in the assembly of complex I, which triggers damage to the mitochondria, eventual cell-proliferation arrest, and death (Wilson et al., 2020). Rett syndrome (RTT) is a neurodevelopmental disorder caused by mutations in methyl-Cpg-binding protein 2 (MECP2) on the X chromosome. The main symptoms include loss of language function, abnormal gait, and repetitive stereotyped movements. Significant reductions in cytochrome c oxidase subunit I levels and the levels of complexes II, III, and IV, mitochondrial respiratory chain enzyme activity, and glutathione levels have been detected in both transcript and protein level studies of brain cells from patients with RTT, suggesting that mitochondrial energy production was severely impaired in these patients (Gold et al., 2014; Müller, 2019).

### Abnormal mitochondrial Ca^2+^ inward flow and mitochondrial permeability transfer pore regulation in neurodevelopmental disorders

**Figure 4:**
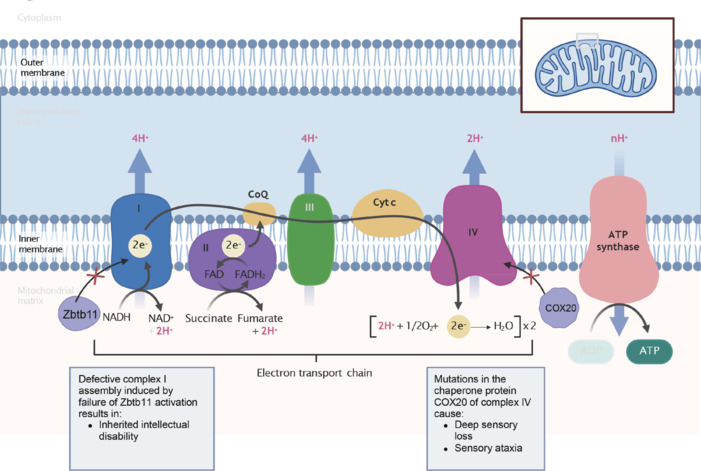
Abnormal oxidative phosphorylation in neurodevelopmental disorders. FADH and NADH produced during the tricarboxylic acid cycle transfer electrons from the reduction products to the electron transport chain in the inner mitochondrial membrane. Electron transport chain consists of four oxidative phosphorylation complexes (I–IV). Electrons pass through four complexes at a time and combine with oxygen at complex IV to form water. Two proteases, CoQ and cytochrome c are responsible for electron transfer between the complexes. Finally, the energy stored by the electrochemical gradient is used to generate ATP in the presence of complex V. The inactivation of Zbtb11-induced complex I abnormalities was identified in familial intellectual disability. In addition, abnormalities in COX20, the chaperone protein of complex protein IV, induce progressive deep sensory loss and sensory ataxia. Created with BioRender.com. ADP: Adenosine Diphosphate; ATP: adenosine triphosphate; ATP synthase: adenosine triphosphate synthetase; CoQ: coenzyme Q; COX20: cytochrome c oxidase assembly protein 20; Cut c: cytochrome c; FAD: flavin adenine dinucleotide; FADH_2_: reduced flavin adenine dinucleotide; NAD⁺: nicotinamide adenine dinucleotide (oxidized form); NADH: nicotinamide adenine dinucleotide (reduced form); Zbtb11: zinc finger and BTB domain-containing protein 11.

In **Figure 4**, elevated Ca^2+^ prevents mitochondrial movement in neurons both in a paracrine and retrograde manner. Mitochondrial arrest is caused by microtubule detachment or by inducing conformational changes in mitochondrial Rho GTPase 1 by preventing motor-adapter interactions (Schwarz, 2013). Mitochondrial calcium homeostasis is a tightly controlled process. Mitochondrial calcium uptake 2 is a major component of the mitochondrial calcium monotransporter complex, and its purist truncation mutations are associated with severe cognitive impairment, spasticity, and NDD with white matter involvement. Patient-derived mitochondrial calcium uptake 2-deficient cells are shown to impair mitochondrial calcium homeostasis, triggering increased mitochondrial sensitivity to oxidative stress and aberrant modulation of mitochondrial endomembrane potential (Shamseldin et al., 2017). Solute carrier family 25 member 12, a susceptibility gene for ASD encoding the mitochondrial aspartate/glutamate carrier, participates in the aspartate/malate-reducing nicotinamide adenine dinucleotide shuttle and is involved in physiological activation of calcium ions. Excess calcium ion levels are responsible for promoting glutamate carrier activity in the autistic brain, supporting oxidative phosphorylation and ATP production, mitochondrial metabolism, and greater oxidative stress (Palmieri et al., 2010; Napolioni et al., 2011).

## Mitochondria as a Therapeutic Target for Neurodevelopmental Disorders

Drugs acting on mitochondria to treat NDDs are detailed in **Table 2**.

**Table 2:** Drugs acting on mitochondria to treat NDDs

Mechanism of action	Clinical syndrome	Drug	Administration method	Efficacy	Side effect
Proteins related to neurokinesis and fusion (Drp1, FIS1)	ASD	BT	Oral	Improve certain symptoms (e.g., social behavior, anxiety, and repetitive behaviors) in ASD patients	Gastrointestinal reactions
Regulation of mitochondrial bioenergy production by activating PGC-1 α	Fragile X syndrome, RTT	Igfbp2	Viral vector, intracerebral injection, or nasal administration	Igfbp2 gene therapy can improve social behavior, cognitive function, and anxiety in FXS mice. Currently in early clinical trial stages with limited large-scale clinical data.	Immune reactions
	Intellectual loss of children with mitochondrial dysfunction caused by fluoride	RSV	Oral	Low absorption rate, and lack of clinical trials	Gastrointestinal reactions
	ASD	PEA	Oral, liquid	Improve social interaction and anxiety in ASD patients but have limited effects on repetitive behaviors	Gastrointestinal reactions and central nervous system side effects
Correction of mitochondrial oxidative phosphorylation chain enzyme defect	Fragile X syndrome	Alpha tocopherol	Oral	Improve behavioral symptoms (e.g., anxiety and social impairment)	Gastrointestinal reactions, vitamin E overdose, and increased bleeding risk
	RTT	Omega-3 polyunsaturated fatty acids	Oral	Improve motor function and behavior	Gastrointestinal reactions and increased bleeding risk
	ASD	Methylene blue and N-acetylcysteine	Oral, intravenous injection	Reduce levels of oxidative stress markers and improve cognitive function	Gastrointestinal reactions and skin reactions
	DS	Coenzyme Q (10), acetyl L-carnitine, α-lipoic acid and ascorbic acid	Oral, intravenous injection	Reduce levels of oxidative stress markers (e.g., malondialdehyde MDA), improve cell function, and help improve cognitive function and behavioral symptoms	Gastrointestinal reactions, central nervous system side effects, skin allergies, and increased risk of kidney stones
	ASD	APT	Oral, intravenous injection	Improves anxiety, social impairment, and repetitive behaviors	Gastrointestinal reactions, central nervous system side effects, and immune system effects
	ADHD	MPH	Oral, transdermal patch	Significantly improve inattention, distractibility, and task completion ability in ADHD patients, regulate neurotransmitter levels, and reduce impulsive behavior and hyperactivity	Gastrointestinal reactions, central nervous system side effects, skin allergies, cardiovascular side effects, and growth retardation
	RTT	5-HT7R	Oral, intravenous injection, transdermal patch	Improve social behavior and anxiety in RTT models	Gastrointestinal reactions, central nervous system side effects, skin allergies, and cardiovascular side effects
	RTT	Bacterial protein CNF1	Viral vector, intracerebral injection, or nasal administration	Significantly reduce oxidative stress markers (e.g., MDA) and inflammatory cytokines (e.g., TNF-α, IL-6) in RTT models	Immune reactions and local tissue damage
Antioxidant and targeted activation of mitochondrial autophagy pathway	Koolen-de Vries syndrome	13-cis retinoic acid	Oral, topical administration	Improve cognitive function and behavioral symptoms in neurodevelopmental disorder models	Skin side effects, gastrointestinal discomfort, central nervous system side effects, and skeletal and muscular side effects
	ASD and schizophrenia	New acyl carnitine ester	Oral, intravenous injection, transdermal administration	Improve hallucinations, delusions, and cognitive impairments	Skin side effects, gastrointestinal discomfort, and central nervous system side effects
	DS	Plant polyphenols	Oral	Significantly improve attention, learning ability, and social behavior in DS patients	Gastrointestinal discomfort
Targeted promotion of mitochondrial biosynthesis	Cerebral palsy	Resveratrol	Oral	Low absorption rate and lack of clinical trials	Gastrointestinal reactions

This table provides a comprehensive overview of various drugs targeting mitochondrial dysfunction in NDDs. These drugs are categorized based on their mechanisms of action, including regulation of mitochondrial dynamics (e.g., fusion and fission), activation of mitochondrial biogenesis through PGC-1α, correction of oxidative phosphorylation defects, and targeted activation of mitochondrial autophagy. Each entry includes the clinical syndrome targeted, the specific drug, its administration method, efficacy, and potential side effects. 5-HT7R: 5-hydroxytryptamine receptor 7; ADHD: attention-deficit/hyperactivity disorder; ASD: autism spectrum disorder; ATP: adenosine triphosphate; BT: baclofen; CNF1: cytotoxic necrotizing factor 1; DS: down syndrome; FIS1: mitochondrial fission 1 protein; FXS: fragile X syndrome; Igfbp2: insulin-like growth factor-binding protein 2; IL-6: interleukin-6; MDA: malondialdehyde; MPH: methylphenidate; NDDs: neurodevelopmental disorders; PEA: palmitoylethanolamide; PGC-1α: peroxisome proliferator-activated receptor-gamma co-activator 1α; RTT: Rett syndrome; RSV: resveratrol; TNF-α: tumor necrosis factor-alpha.

### Regulation of mitochondrial permeability and fusion kinetics

Regulating mitochondrial permeability and fusion dynamics is an effective approach to treating NDDs. Butyrate (BT), a short-chain fatty acid primarily produced by the gut microbiome, has emerged as a promising candidate for this purpose. BT positively modulates mitochondrial function by enhancing oxidative phosphorylation and fatty acid oxidation, thereby exerting neuroprotective effects. It has been shown to increase the expression of proteins involved in mitochondrial fission (e.g., PINK1, DRP1, FIS1) and physiological stress response (e.g., UCP2, mTOR, HIF1α, PGC1α), as well as those associated with cognition and behavior (e.g., CREB1, CamKinase II). These findings suggest that BT can enhance mitochondrial function, particularly in the context of physiological stress and mitochondrial dysfunction.

BT is primarily administered orally, since it is absorbed both passively and actively through specific monocarboxylate transporters. It can also be delivered via intraperitoneal injection, which has been shown to be effective in animal models of neurodevelopmental disorders. In these models, BT has demonstrated therapeutic potential in improving cognitive deficits and behavioral abnormalities associated with conditions such as ASD and Parkinson’s disease. For instance, BT has been shown to rescue ASD-like behaviors and brain pathology in animal models by modulating neurotransmitter gene expression and improving mitochondrial function. However, the clinical application of BT faces several challenges. High concentrations of BT may cause gastrointestinal discomfort, and its effects can be influenced by the microenvironment’s redox state and the underlying mitochondrial function of the cells. Additionally, early postnatal exposure to BT has been linked to colitis in some studies. These potential adverse effects highlight the need for careful dosing and further investigation into the long-term safety of BT.

Despite these challenges, recent research has provided valuable insights into the therapeutic potential of BT. For example, studies have shown that BT can modulate the expression of genes involved in learning, memory, and behavior, such as cAMP response element-binding protein and CamKinase II. Thus, BT may play a role in regulating repetitive and obsessive behaviors associated with ASD. Moreover, BT has been found to improve mitochondrial function in lymphoblastoid cell lines derived from children with ASD, particularly in those with mitochondrial dysfunction. These findings indicate that BT could be a promising treatment option for children with ASD and other NDDs, although additional preclinical and clinical studies are needed to explore its practical implications. Butyrate holds significant promise for treating NDDs by regulating mitochondrial function and modulating gene expression related to cognition and behavior. While its administration through oral or injection routes has shown therapeutic potential in animal models, the potential for gastrointestinal side effects and the need for precise dosing highlight the necessity for further research. Future studies should focus on optimizing BT’s delivery methods, elucidating its long-term effects, and exploring its potential as a targeted therapy for NDDs (Rose et al., 2018).

### Regulation of mitochondrial bioenergetics by activation of peroxisome proliferator-activated receptor gamma coactivator 1-alpha and others

The regulation of mitochondrial bioenergetics through the activation of PGC-1α and other related pathways has emerged as a promising strategy for addressing mitochondrial dysfunction in NDDs. Postmortem tissues and animal models of ASD have shown an increased number of microglia and astrocytes. Recent studies have indicated that astrocytes play a crucial role in synapse formation and dendritic spine regulation, with synaptic plasticity being dependent on mitochondrial biogenesis in these cells (Blanco-Suarez et al., 2017; Koeppen et al., 2018). The key regulator of this process is the transient upregulation of PGC-1α, which orchestrates mitochondrial biogenesis. In the absence of astrocytic PGC-1α, astrocyte proliferation is stimulated, further exacerbating neuroinflammation. Moreover, the release of insulin-like growth factor-binding protein 2 (Igfbp2), an inhibitor of insulin-like growth factor secretion, has been shown to reverse neurite growth inhibition by blocking bone morphogenetic protein (BMP) signaling in astrocytes associated with fragile X syndrome and RTT (Zehnder et al., 2021). In addition, increased release of Igfbp2 reverses the inhibitory effect of neurite growth by blocking BMP signaling in astrocytes of patients with fragile X syndrome and RTT (Caldwell et al., 2022). The sirtuin 1-specific activator resveratrol averts developmental fluoride neurotoxicity by activating the sirtuin 1-dependent PGC-1α/NRF1/TFAM signaling pathway, rescuing children from intellectual loss due to fluoride-induced mitochondrial dysfunction (Zhao et al., 2020). Palmitoylethanolamide (PEA) has been shown to ameliorate mitochondrial dysfunction by restoring the hippocampal brain-derived neurotrophic factor signaling pathway (Cristiano et al., 2018). In clinical studies, these compounds have been administered through various routes, including oral and intraperitoneal injection, and have shown varying efficacy and safety profiles. Resveratrol, for example, is typically administered orally and has been shown to cross the blood–brain barrier, exerting neuroprotective effects. However, its bioavailability remains a challenge, and the high doses required to achieve therapeutic effects could lead to gastrointestinal side effects. PEA, on the other hand, has been used in both animal and human studies to modulate inflammation and mitochondrial function, with fewer reported adverse effects. Its efficacy in restoring brain-derived neurotrophic factor levels and improving mitochondrial function in the hippocampus highlights its potential as a therapeutic agent for NDDs. Despite these promising findings, the clinical application of these compounds requires further investigation. The long-term safety and optimal dosing strategies for resveratrol and PEA are yet to be fully established. Additionally, the heterogeneity of NDDs necessitates personalized approaches, and future studies should focus on identifying biomarkers to predict therapeutic response and minimize adverse effects. The integration of multi-omics technologies and patient-specific induced pluripotent stem cell (iPSC) models could provide valuable insights into the underlying mechanisms and facilitate the development of targeted therapies.

The activation of PGC-1α and related pathways offers a promising avenue for enhancing mitochondrial bioenergetics and improving neurodevelopmental outcomes. While compounds such as resveratrol and PEA have shown therapeutic potential in preclinical studies, further research is needed to optimize their clinical application and address the challenges associated with bioavailability and long-term safety.

### Correction of mitochondrial oxidative phosphorylation chain enzyme defects

Correction of mitochondrial oxidative phosphorylation, streptokinase defects, and oxidative stress can serve as new therapeutic targets in NDD. The role of mitochondrial oxidative phosphorylation system dysfunction and the resulting oxidative stress in the pathogenesis of a variety of NDDs is supported by evidence. Thus, targeting the alleviation of oxidative stress and energy deficits and thereby ameliorating the associated clinical phenotypes is considered to be an attractive therapeutic strategy. One promising approach for fragile X syndrome is pharmacological treatment with alpha-tocopherol, an antioxidant/free radical scavenger, which ameliorates the behavioral and learning deficits in patients with the syndrome (Osakada et al., 2003). In the treatment of RTT, exogenous compensatory omega-3 polyunsaturated fatty acid administration based on the regulation of oxidative stress has been shown to reverse some of the pathophysiological features. Augmentation of ROS production and oxidative stress can exacerbate the severity of the condition in autism and Down syndrome. Methylene blue and N-acetylcysteine have been proposed to be effective for autism treatment. Their therapeutic mechanisms involve neuroprotection by bypassing complex I/III to cytochrome c, increasing ATP production, and decreasing ROS production (Ghanizadeh et al., 2013). Mitochondria-targeting nutrients used to alleviate oxidative stress and exerting a positive effect on mitochondrial function are widely used in the treatment of Down syndrome, such as coenzyme Q (Onishi et al., 2021), acetyl l-carnitine, alpha-lipoic acid, and ascorbic acid (Valenti et al., 2014). Methylphenidate, a central nervous system stimulant, is widely used in the treatment of ADHD. Methylphenidate ameliorates impairment of enzyme activity in the Krebs cycle and electron transport chain, increasing ATP and Na levels as well as K-ATPase activity, thereby maintaining brain energy homeostasis (Foschiera et al., 2022). Brain serotonin receptor seven can be targeted for the treatment of RTT by repairing the oxidative phosphorylation disorders caused by damage to the mitochondrial respiratory chain (Valenti et al., 2017). The bacterial protein cytotoxic necrotizing factor 1 has been used to treat RTT in mice. Reactivation of the respiratory chain complex and prevention of hydrogen oxide overproduction in the brain were observed in the brains of RTT mice treated with cytotoxic necrotizing factor 1 (De Filippis et al., 2015). However, these studies mostly focused on the regulation of damaged protein complexes in the respiratory chain and the carriers involved in transport of electrons while ignoring the clearance of the generated ROS. Scavenging ROS in cells can be further explored to reduce cell damage in future studies. Clinically, these therapeutic agents are administered through various routes, including oral and intravenous delivery. While they show promise in preclinical models, their long-term safety and efficacy in humans require further investigation. For example, N-acetylcysteine is generally well-tolerated but may cause gastrointestinal side effects at high doses. Similarly, coenzyme Q10 has shown benefits in mitochondrial disorders but requires optimization of dosing and delivery methods to enhance bioavailability.

Overall, correcting mitochondrial oxidative phosphorylation chain enzyme defects represents a multifaceted approach to addressing mitochondrial dysfunction in NDDs. Future research should focus on optimizing therapeutic strategies, exploring combination therapies, and conducting larger clinical trials to establish the safety and efficacy of these interventions in diverse patient populations.

### Targeted activation of the mitochondrial autophagy pathway

In Koolen-de Vries syndrome, where mitochondrial clearance is impaired due to abnormal autophagy, the Food and Drug Administration-approved drug 13-cis retinoic acid has shown promise in rescuing mitochondrial activity. This drug facilitates the fusion of autophagosomes with lysosomes, thereby alleviating the accumulation of damaged mitochondria and ROS in neurons and cardiac tissues. This ultimately leads to the therapeutic reversal of neurobehavioral abnormalities in KANSL1 heterozygous mice (Li et al., 2022; Linda et al., 2022). The drug is administered orally and has demonstrated significant efficacy in restoring autophagic flux and improving mitochondrial health. However, its clinical application requires careful consideration due to the potential teratogenic side effects observed in other contexts. Future studies should explore safer analogs or related compounds, such as all-trans RA or 9-cis RA, which may offer similar therapeutic benefits with fewer adverse effects. In the treatment of ASD and schizophrenia, acylcarnitine has emerged as a potential neuroprotective agent. Acylcarnitine functions as an antioxidant and a major regulator of the mitochondrial autophagy pathway, exerting beneficial effects on mitochondrial dynamics and overall neuronal health. Acylcarnitine is typically administered orally and has shown promising results in preclinical models, although its long-term safety and efficacy in humans remain to be determined through larger clinical trials (Moos et al., 2016). Antipurinergic treatment has also been explored for its potential to restore the activity of aberrant protein complexes in the mitochondrial respiratory chain. This treatment rescues fragile X protein expression in the brain, leading to the alleviation of social interaction deficits and behavioral limitations seen in ASD. APT is generally administered through intravenous or intraperitoneal injection in animal models, but its translation to clinical practice will require further investigation into dosing, delivery methods, and potential side effects (Naviaux et al., 2013). Plant polyphenols have also garnered attention for their ability to ameliorate energy deficits in Down syndrome by modulating key metabolic pathways, including acetyl coenzyme A/NADPH, lipid oxidation, and homocysteine metabolism. These compounds are effective in scavenging mitochondrial ROS and initiating antioxidant programs to maintain mitochondrial homeostasis. Polyphenols are typically administered orally and have shown minimal adverse effects in preclinical studies. However, their bioavailability and long-term impact on human health need to be further evaluated in clinical trials (Vacca et al., 2016).

These therapeutic approaches targeting mitochondrial dysfunction and oxidative stress hold substantial promise for improving outcomes in NDDs. However, their clinical application requires careful consideration of dosing, delivery methods, and potential adverse effects. Future research should focus on optimizing these treatments, exploring combination therapies, and conducting larger clinical trials to establish their safety and efficacy in diverse patient populations.

## Animal Models of Neurodevelopmental Disorders

NDDs are a group of diseases that develop during a child’s development and have a lifelong impact on children’s health. They include intellectual disabilities, communication disorders, ASD, ADHD, tic disorders, specific learning disabilities, and other NDDs (Bonetti et al., 2024). A variety of animal models have been developed for these diseases.

### Animal models of autism

ASD is a group of behavioral-defined NDDs characterized by three core symptom areas: impairments in social interaction, communication abnormalities, and restricted and repetitive behavior patterns (Mamun et al., 2025). The growing number of cases of ASD and the high costs of treatment and care have made this disease a major public health problem. The etiology of ASD is unknown, and multiple factors such as genetic vulnerability and environmental factors may contribute to the autism phenotype. Different preclinical research models, including chemically induced models, genetically engineered models, and spontaneous models, have been developed to study behavioral phenotypes and underlying pathophysiology of ASD and develop new therapies for ASD. A variety of animal models have been constructed. These models can be roughly divided into three categories: genetic animal models, environmental factor–induced animal models, and idiopathic animal models. The most commonly used autism models include six commonly used genetic models (*Ube3a*, *Pten*, *Nlgn3*, *Shank3*, *MECP2*, and *Fmr1* mutant or knockout mice) and three chemically induced models (valproic acid [VPA], lipopolysaccharide [LPS], and polyinosine). Many mouse models with mutations in genes at risk for ASD have been developed and shown to have altered behaviors associated with ASDs (Assimopoulos et al., 2022; Panzenhagen et al., 2022).

The prevalence of neurodevelopmental disorders was 6–10 times higher in children with a history of prenatal VPA exposure, with ASD being the most common manifestation of neurodevelopmental disorders at 6 years of age in children with prenatal VPA exposure (Bromley et al., 2013). In animal experiments, rats or mice exposed to VPA prenatally are classic animal models for ASD research and are widely used in studies to explore potential therapeutic drugs for ASD. Studies have shown that the social ability, social preferences, repetitive behavior, and exploratory behavior of mice exposed to VPA prenatally are generally impaired (Silva et al., 2025). In terms of molecular mechanism, the researchers conducted a preliminary investigation on the molecular mechanisms of VPA in embryonic development and early induction of ASD, and found that VPA exposure regulates some signaling pathways, such as histone hyperacetylation, the wingless/integrated signaling pathway, the extracellular signal-regulated kinase–p21 signaling pathway, and 1-amino, butyric acid levels in the brain, thereby affecting brain development and neural networks maturation, ultimately leading to ASD-like behavioral phenotypes in offspring (Fang et al., 2025).

BTBR mice were originally bred by Dunn of Columbia University using mice carrying the short-tailed gene and mice carrying the cluster gene mutation, resulting in continuous inbreeding (Varghese et al., 2017). The abnormal behavior of BTBR mice is mainly caused by the three core solo glycoside polymorphisms of the Kmo gene encoding kynurenine 3-monooxygenase. BTBR mice can adequately simulate the core symptoms of clinical ASD patients, so they are widely used in studies on the pathological mechanisms underlying ASD and the screening of drug candidates. BTBR mice show obvious social dysfunction such as low social motivation and lack of social ability in the three-box social ability experiment. In the direct social interaction experiment, BTBR mice showed significantly reduced exploratory social behaviors such as sniffing and following. In the Ultrasonic Vocalization test, the BTBR mice emitted unusually high-frequency, high-amplitude ultrasound vocalizations when they left the female mouse’s cage. The researchers believed that this behavior is similar to the loud crying of children with ASD when they are forced to be separated from their mothers. In contrast, the number of ultrasonic waves emitted by adult BTBR mice when confronted with the smell of unfamiliar mice decreased, which is similar to the language communication impairment of human ASD patients. In addition, the study also showed that BTBR mice exhibited increased repetitive behaviors, such as solitary living, digging, grooming, and beading (Bridi et al., 2025). The anatomical features of BTBR mice are also consistent with those of ASD patients. For example, in BTBR mice, the organic lesions of the central nervous system are mainly concentrated in the dysplasia of the cortex, corpus callosum, and hippocampus. The most significant neuroanatomical features are 100% loss of the corpus callosum and severe reduction in hippocampal associations. These features are similar to the clinical manifestations of ASD patients (Chen et al., 2025c).

Studies on the maternal immune activation (MIA) model originated from a large-scale epidemiological investigation of the pathogenic factors of ASD. Infection during pregnancy, especially in the first three months, is closely related to the risk of ASD in offspring. In animal experiments, MIA models can be successfully constructed by exposing pregnant female mice or rats to factors such as polyinosinic-polycytidylic acid (Poly I:C), LPS, simulated viruses, and bacteria. These factors activate the immune system by inducing maternal immune responses (Santoni and Pistis, 2024). In the Ultrasonic Vocalization test, the ultrasonic vocal frequency of MIA offspring mice decreased in the face of different social stimuli, indicating the presence of language communication disorders in the MIA offspring mice. In the three-compartment social-ability test, the social preference of MIA offspring mice was significantly reduced, indicating the presence of social communication disorders in MIA offspring mice. In the beading and grooming experiments, MIA offspring mice showed greater repeated stereotyped behaviors. These results suggest that the offspring mice of the poly I:C immune activation model exhibited very similar behavioral characteristics to those of ASD patients, such as social communication disorders, grooming, repetition, nesting and stereotyped behaviors (Naviaux et al., 2014).

### Animal models of schizophrenia

Schizophrenia is a highly destructive and complex psychiatric disease that is accompanied by various positive and negative symptoms and cognitive impairment, all of which place a heavy burden on society. This disease involves complex interactions between genetic and environmental factors during neurodevelopment; as a result, elucidation of the etiology of schizophrenia and the development of treatment approaches remains quite challenging. Therefore, the establishment of appropriate animal models can help people understand the neurobiological basis of schizophrenia caused by various pathogenic factors and provide clues and theoretical basis for finding new treatment methods. At present, the main modeling methods include neurodevelopmental models, drug-induced models, and genetic mouse models.

For the establishment of neurodevelopmental models, the most widely used methods include prenatal injection of methylazoxymethanol, post-weaning social isolation, “pregnancy infection model,” and the second-strike hypothesis. Methylazoxymethanol is an anti-mitotic agent that methylates DNA and specifically acts on the proliferation of neuroblasts without affecting glial cells. Injection of methylazoxymethanol at different stages of pregnancy can cause different neurodevelopmental abnormalities, and administration on the 17^th^ day of gestation in rats is a common method for constructing animal models of schizophrenia. In the brain of the offspring, structural changes include reduced medial prefrontal volume, increased lateral and third ventricles, a decrease in intermediate nerve cell markers in marginal and cortical areas, and an increase in dopamine nerve cell activity in the ventral tegmental area. Behavioral phenotypes include increased motor response to the dopamine pathway agonist amphetamine, decreased social activity, deficits in sensorimotor gating, memory impairment, and increased anxiety (Takahashi et al., 2019; Sonnenschein and Grace, 2020).

Post-weaning social isolation is based on the fact that rodents display a well-defined social structure and hierarchy in the population, and deprivation of this trait can have important effects on their neurodevelopment. Therefore, social isolation of young mice from weaning can lead to a range of behavioral deficits in adulthood, including increased levels of spontaneous movement, responsiveness to novelty, augmentation, sensorimotor gating disorders, cognitive impairment, and increased anxiety and aggression, which are very similar to the core symptoms of schizophrenia (Desbonnet et al., 2022; McGrath and Briand, 2022). Antipsychotic drugs such as haloperidol, olanzapine, and risperidone are known to reverse the increase in spontaneous movement caused by social isolation. Thus, the social isolation-induced schizophrenia model after weaning has a high predictive validity, and it can be used to test the efficacy of drugs to reverse positive symptoms of schizophrenia. At the same time, the model also has good structural validity. The model animals showed reduced frontal lobe volume, dendritic spine density, and reductions in hippocampal and parvalbumin-positive intermediate nerve cells. These histological abnormalities can also be observed in patients with schizophrenia (Hamieh et al., 2021; Moghadam et al., 2021).

Another model-building strategy is the use of polyinosine polycytidine to induce MIA in pregnant rodents. MIA has been suggested to induce long-term immune changes in the brains of rodent and primate offspring, which may underlie epigenetic changes that mediate behavioral and brain structural changes in animals similar to the pathological changes in schizophrenia (Vlasova et al., 2021; Xu et al., 2024). Animal models of drug-induced schizophrenia are widely used, and the principle of model construction is based on the hyperactivity of dopaminergic pathways projected to marginal regions in etiology (Białoń and Wąsik, 2022), and the reduced function of N-methyl-d-aspartate receptors in glutamate receptors (Nakazawa and Sapkota, 2020). Thus, drugs commonly used in animal models are dopamine pathway agonists (e.g., amphetamines, apomorphine) and non-competitive N-methyl-D-aspartate receptor antagonists (e.g., phencyclohexidine, ketamine, diazocycline, and pyrifoxine). Among them, amphetamine-induced psychiatric symptoms include hypermovement, spatial working memory impairment, sensorimotor gating deficits, and potential inhibitory deficits (Arroyo-García et al., 2021; Pedrazzi et al., 2024). Genetic factors have been the focus of research in the etiology of schizophrenia, and are the basis for many mouse models of schizophrenia. Many candidate genes are associated with an increased risk of schizophrenia, and most of them are associated with nerve cell plasticity, glutaminergic or dopaminergic neurotransmitters, and synaptogenesis (Lv et al., 2024). Identifying candidate genes is the first step in building a complex animal model of schizophrenia. The etiopathology of schizophrenia is likely to involve more than one gene, including complex gene-environment interactions and gene-gene interactions. The schizophrenia defect 1 gene (disrupted-in-schizophrenia 1, *DISC1*) primarily encodes the synaptic protein DISC1, which is expressed early in development. Genetic analysis showed that the *DISC1* gene is considered to be a highly associated genetic factor in schizophrenia in humans (Rittenhouse et al., 2021). DISC1-induced and/or partial loss of function in mice resulted in deficits in recognizing memory, social, and anxious behaviors, as well as sensorimotor gating abnormalities, which are typical behavioral symptoms of schizophrenia (Park et al., 2025). 22q11.2 deletion syndrome is a genetic syndrome caused by the deletion of the 22q11. 2 chromosome. A large amount of genetic evidence suggests that the deletion of 22q11.2 is related to schizophrenia in humans (Rummell et al., 2023). Mutations in the cell adhesion molecule neuregulin-1 and its receptor erythroblastic leukemia viral oncogene homolog 4 also increase the risk of schizophrenia (Rodríguez-Prieto et al., 2024).

### Animal models of attention deficit hyperactivity disorder

ADHD is a common neurodevelopmental disorder in childhood. The global incidence rate of ADHD is approximately 7.2%, and about 50% of the patients’ symptoms will persist into adulthood. The typical clinical manifestations of ADHD are hyperactivity, impulsivity, and inattention. Moreover, individuals with ADHD often show conduct disorder, tic disorder, learning disorder, speech and language developmental disorders, and oppositional defiant disorder. In basic research on ADHD, animal models are important to meet the requirements of rapid invasive operation and strict hypothesis testing, the primary conditions for disease research. The existing ADHD models include genetic models, environmental induction models, and neurodevelopmental disorder models. Genetic models mainly target genes associated with the risk of developing ADHD. The dopamine transporter in the presynaptic membrane is primarily involved in reuptake of dopamine from the synaptic gap into the presynaptic membrane vesicles for storage, control of extracellular dopamine levels, and intracellular dopamine storage. It is also the main substance that terminates the action of dopamine (Suzuki et al., 2024). Dopamine transporter-knockout mice showed clinical symptoms and behavioral manifestations consistent with ADHD, with good face validity. In the dopamine transporter–knockout mouse model, the dopamine released in the synaptic space could not be cleared in time and spread outside of the cell, which severely reduced the utilization and storage of dopamine, resulting in a nearly 13-fold decrease in the content of dopamine in the striatum (Mallien et al., 2022). Spontaneously hypertensive rats exhibit typical clinical symptoms of ADHD such as spontaneous hyperactivity, impulsivity, and learning cognitive impairment, and are currently the most widely used animal model of ADHD (Sable et al., 2025). However, their use is associated with some controversy. Hypertension is a confounding factor, since the symptoms of ADHD patients do not include hypertension. Although spontaneously hypertensive rats do not show hypertension before 10 weeks of age, the testing schedule is delayed. In addition, high blood pressure can lead to dysfunction, brain damage, and cognitive impairment. During the experimental process, the interference of high blood on the experimental results is unavoidable (Santisteban et al., 2023; Tchekalarova et al., 2024). Synapse-associated protein 25 is a type of SNARE protein located in the plasma membrane cytoplasmic surface binding protein receptor. SNARE proteins are a large family of transmembrane proteins located on the membrane vesicle, which can initiate vesicle fusion, participate in the activation and fusion process of protein and membrane transport regulation and non-regulatory vesicle exocytosis activity, and regulate transmitter release (Cheng et al., 2025). Mice with the synapse-associated protein 25 semi-dominant mutation deletion exhibited neurodevelopmental delays in the delayed reinforcement task, and behavioral deficits, including hyperactivity, impulsivity, and inhibition, were impaired, but did not exhibit attention deficits that did not fully satisfy the face validity of animal models of ADHD (Regan et al., 2022).

A previous study has shown that maternal stress during pregnancy, hypoxia during childbirth, and exposure to alcohol, nicotine, and methamphetamine during prenatal or developmental periods can significantly increase the probability of ADHD in offspring (Ochozková et al., 2021). Environmental induction models mainly include prenatal alcohol exposure (Alger et al., 2021), prenatal nicotine exposure (Jang et al., 2020), prenatal methamphetamine exposure (Čechová et al., 2023), and developmental pyrethroid pesticide deltamethrin exposure (Curtis et al., 2024). Animals exposed to alcohol before birth exhibited ADHD symptoms such as attention deficit, hyperactivity, and impulsivity from infancy. In addition, perinatal alcohol exposure also led to visual-spatial deficits, another manifestation of ADHD. Animals exposed to alcohol prenatally also exhibited attention deficits and learning disabilities in the Morris Water Maze, spiral arm maze, and T-maze, demonstrating good face validity. Consistent with the findings for patients with ADHD, the fetal alcohol animal model showed fewer nerve cells in multiple areas of the brain, including the prefrontal cortex, cerebellar cortex, and other regions. ADHD is characterized by low dopaminergic activity in several brain regions, such as midbrain limbic, midbrain cortex, and substantia nigra striatum. Studies have shown that the fetal alcohol animal models also show low dopamine function, indicating that this model has good structural validity (Ju et al., 2020; Sharma et al., 2021). Prenatal exposure to nicotine, including maternal active and passive smoking, can lead to a significant increase in the risk of developing ADHD in children after birth, as manifested by impaired reading ability, language impairment, and impaired memory. Studies have shown that prenatal exposure to nicotine in animals can cause hyperactivity, impulsivity, and attention and memory impairments in offspring, in addition to non-ADHD manifestations such as growth retardation, poor adaptability, and anxiety. Rats exposed to nicotine during pregnancy showed memory deficits in the spiral arm maze, impaired memory and impulsivity in the Five-Choice Serial Reaction Time Task, showing face validity, but also showing symptoms that are not part of ADHD. Children and adolescents exposed to nicotine during pregnancy experienced reductions in the volume of the cerebellum, frontal lobe, and lateral ventricle systems, and reductions in the volume of the corpus callosum and cerebellum were also observed in children with ADHD, indicating an intrinsic connection between the two (Contreras et al., 2022; Zhou et al., 2024).

Methamphetamine (METH) is a psychostimulant whose mechanism of action is a sharp increase in serotonin and dopamine levels, causing damage to nerve cells and leading to long-term damage to dopaminergic and serotonergic axonal end points in the hippocampus, striatum, and prefrontal cortex. Prenatal exposure to METH causes hyperactivity symptoms in rats and learning and memory impairments associated with the hippocampal glutamate system. It also impairs the recognition and memory of rats, as well as their ability to concentrate. The object localization test assesses spatial memory and spatial discrimination. The object localization test assesses spatial, memory, and spatial discrimination. Studies have shown that rats exposed to METH prenatally exhibited hyperactivity in the object localization test but no impulsive symptoms and limited facial validity. Prenatal exposure to METH can lead to changes in brain morphology and brain metabolism, increase the basal level of dopamine in the nucleus accumbens of rats, and have limited structural validity. Owing to damage to the dopaminergic end point, the effects of psychostimulants are very limited and cannot reflect their predictive validity (Ochozková et al., 2019, 2021). Childhood exposure to pyrethroids is closely associated with the onset of ADHD. Studies have shown that mice exposed to the pyrethroid pesticide deltamethrin during development exhibit ADHD clinical symptoms such as hyperactivity, impulsivity, and inattention, as well as memory deficits and good facial validity in the Y-maze. From the perspective of the mechanism of influence, these behavioral changes are caused by disruption of the dopamine system, including increased levels of dopamine transporters in the brain, reduced levels of dopamine in synapses, and increased levels of D1 dopamine receptors, which is consistent with the reduction in dopamine levels in ADHD patients, with structural validity. ADHD-like symptoms in mice exposed to deltamethrin can be controlled by methylphenidate hydrochloride, which reflects its predictive validity. The mouse model exposed to deltamethrin during development can better meet the three validities of the ADHD animal model, but it is still uncertain which period of development and what dose and method of administration can harvest better model mice. The corresponding modeling method is worth further exploration (Vester et al., 2020; Xi et al., 2022).

In addition to genetic and environmental influences, abnormalities in brain structure and function are also important factors in the pathogenesis of ADHD. Models of neurodevelopmental disorders include cerebellar growth retardation models, neonatal hypoxia rat models, maternal stress mouse models, neonatal 6-hydroxydopamine-impaired rat models, and neonatal caries animal models of X-ray radiation. Cerebellar volume is significantly reduced in children with ADHD, and cerebellar growth retardation plays an important role in ADHD. Various drugs have been used to treat cerebellar damage, such as methoxymethanol on the 4^th^ day after birth, which causes hyperactivity and subsequent conversion to mild hyperactivity, but amphetamine treatment aggravates hyperactivity in rats and lacks predictive validity. On postnatal day 7, rats treated with dexamethasone presented a significant decrease in cerebellar volume and mild hyperactivity in open-field experiments, but the findings were not validated. Although ADHD patients show a decrease in cerebellar volume, there is still much uncertainty about which drug to use to induce (de la Peña et al., 2018; Rahi and Kumar, 2021). Neonatal hypoxia has been implicated in many neurodevelopmental disorders, including ADHD. Studies have shown that neonatal hypoxic rats exhibit ADHD-like behaviors such as learning, memory impairment and hyperactivity. In the rat brain, the content of dopamine in the striatum decreases, which is consistent with the pathological state of children with ADHD, reflecting structural validity. The hyperactivity of neonatal hypoxic rats can be alleviated by the psychostimulant amphetamine, reflecting its predictive validity. Although the neonatal hypoxic rat model has a certain surface area, structure and predictive validity, it does not show all the core symptoms of ADHD. The surface validity did not fully match. The duration and degree of hypoxia during modeling need further consideration (Hamdy et al., 2020; Miguel et al., 2022). Maternal stress-induced mice presented symptoms similar to those of ADHD mice. When adult mice are under stress during pregnancy, their offspring exhibit hyperactivity but not impulsivity or inattentiveness, with limited facial validity. After the mother mouse was stressed, the offspring mice presented reduced striatal dopamine transporter activity and reduced utilization of dopamine transmitters in adulthood, with structural validity. When dopamine antagonists were used, the hyperactivity symptoms in the mice were relieved, indicating that the hyperactivity symptoms in the mice were correlated with dopamine. However, these model mice only exhibited hyperactivity, and other core symptoms of ADHD were not involved. As a model of ADHD, its structural validity and predictive validity are lacking (Bielas et al., 2014; Bronson and Bale, 2014). Neonatal 6-hydroxydopamine-impaired rats are considered potent ADHD model rats because of the selective chemical damage to dopaminergic nerve cells in 5-day-old rats. The rats exhibited hyperactivity and inattention but did not demonstrate impulsivity due to hyperactivity or learning impairment. They exhibited partial face validity in spatial discrimination tests. The rats exhibited hyperactivity and learning impairment along with a decrease in dopamine in the striatum, prefrontal lobe, cortex, septum, midbrain, and amygdala. In rats with 6-hydroxytryptamine lesions, D4 receptor levels are increased in the caudate nucleus, and D4 receptor polymorphisms are closely related to ADHD. In addition, serotonin increased in the rat brain striatum. Dopamine transporter inhibitors did not affect hyperactivity in rats, but D4 receptor antagonists and inhibitors of serotonin transporters significantly reduced hyperactivity, indicating that the increase in serotonin in the striatum was closely related to hyperactivity in rats, with structural validity. The hyperactivity and inattention of neonatal 6-hydroxydopamine-damaged rats could be improved by methylphenidate hydrochloride and amphetamine, and the predictive validity was consistent. The hyperactive performance of a rat model with 6-hydroxytryptamine-dopamine impairment shows partial surface validity but is not comprehensive, and structural validity is also limited (Kantak, 2022; Carreón-Trujillo and Corona, 2024). X-ray irradiation of newborn caries leads to behavioral deficits, including learning and memory deficits. Studies have shown that rats exposed to X-rays after birth exhibit significant hyperactive and impulsive behavior. The reduced number of nerve cells in the hippocampus of the rat brain in this model is thought to be the cause of symptoms, and amphetamine can reduce the behavioral and memory deficits caused by X-ray irradiation in rats. However, many practical problems and other controversies have resulted in the reliability of this model being questioned, so it has not been widely used (Hanbury et al., 2016; Selemon and Begovic, 2020).

## Mitochondrial Deficits in Neuronal Cells and Neurodevelopmental Disorders

The brain is a complex organ that contains not only nerve cells but also a variety of nonnerve cells, such as glial cells (including astrocytes, microglia, and oligodendrocytes), vascular-associated endothelial cells, and pericytes (Bernier et al., 2025). These nonnerve cells play important roles in maintaining brain homeostasis, supporting nerve cell function, and coordinating the formation of neural circuits during development (Chen et al., 2025d; Tamatta et al., 2025). However, studies of NDDs tend to focus on mitochondrial dysfunction in nerve cells, ignoring the potential effects of mitochondrial defects in nonnerve cells (Zehnder et al., 2021; Abhilash et al., 2025). Indeed, mitochondrial dysfunction in nonnerve cells may also have profound effects on neurodevelopment.

### Effects of mitochondrial dysfunction in nonnerve cells on neurodevelopment

#### Astrocytes

Astrocytes are among the ost abundant types of glial cells in the brain, and they play a key role in maintaining the integrity of the blood–brain barrier, providing metabolic support, and regulating synaptic transmission (Rawani et al., 2024; Soylu et al., 2025). Mitochondrial dysfunction may result in impaired energy metabolism in astrocytes, which in turn affects their support for nerve cells. For example, astrocytes support the survival and function of nerve cells by releasing neurotrophic factors, and mitochondrial dysfunction may impair this support (Dave and Pillai, 2022; Wang et al., 2023). In addition, mitochondrial dysfunction in astrocytes may also trigger oxidative stress and inflammatory responses, further disrupting the neurodevelopmental microenvironment (Feng et al., 2024; Mazzantini et al., 2025).

#### Microglia

Microglia are the brain’s immune cells responsible for clearing cellular debris and pathogens and are involved in synaptic pruning (Shu et al., 2025; Zhao et al., 2025). Mitochondrial dysfunction may lead to the activation of microglia, triggering neuroinflammation (Ayyubova and Madhu, 2025; Karayel et al., 2025). Chronic inflammation disrupts the neurodevelopmental microenvironment, affecting nerve cell differentiation and synaptic formation (Gu et al., 2025; Liu and Zhang, 2025). In addition, mitochondrial dysfunction in microglia may further exacerbate nerve cell damage by releasing inflammatory mediators (D’Egidio et al., 2024; Ghosh and Pearse, 2024; Chen et al., 2025a).

#### Endothelial cells and pericytes

Cerebrovascular endothelial cells and pericytes are crucial for maintaining blood–brain barrier function and regulating cerebral blood flow (Hoover et al., 2025; Negri et al., 2025). Mitochondrial dysfunction may result in impaired energy metabolism in these cells, which in turn weakens their barrier function (Gil et al., 2025; Wang et al., 2025e). For example, mitochondrial dysfunction in endothelial cells may lead to vascular leakage, increasing the permeability of the blood‒brain barrier, which in turn triggers an inflammatory response (Payne et al., 2023; Hu et al., 2024). In addition, mitochondrial dysfunction in pericytes may affect their support of blood vessels, leading to abnormal regulation of cerebral blood flow (van Hameren et al., 2023; Morton et al., 2025).

#### Oligodendrocytes

Oligodendrocytes are responsible for the formation and maintenance of myelin sheaths and are essential for the rapid transmission of nerve impulses (Bazzi et al., 2025; Ziar et al., 2025). Mitochondrial dysfunction can lead to abnormal myelination, which in turn affects the normal function of nerve cells. For example, mitochondrial dysfunction in oligodendrocytes can lead to the degeneration of myelin sheaths, affecting the conduction efficiency of nerve cells (Alvarez-Sanchez and Dunn, 2023; López-Muguruza and Matute, 2023).

### Effects of nonnerve cell mitochondrial dysfunction on neurodevelopmental disorders

Mitochondrial dysfunction in nonnerve cells can affect neurodevelopmental disorders through a variety of mechanisms. For example, mitochondrial dysfunction in astrocytes and microglia may trigger oxidative stress and inflammatory responses that disrupt the neurodevelopmental microenvironment (Romagnolo et al., 2024; Toro-Urrego et al., 2024; Wang et al., 2024c). In addition, mitochondrial dysfunction in endothelial and pericyte cells may lead to impaired blood–brain barrier function, increasing the risk of damage to nerve cells (Odeh et al., 2024). Mitochondrial dysfunction in oligodendrocytes may lead to abnormal myelination and affect the transmission of nerve impulses (Cossu et al., 2024; Tsitsikov et al., 2024).

### Research progress and future directions

In recent years, research on the role of mitochondrial dysfunction in NDDs has gradually increased, with a particular focus on the molecular mechanisms and therapeutic strategies involved. However, most of the current research has focused on neuronal cells, whereas investigations of mitochondrial dysfunction in nonneuronal cells are relatively limited. Nonneuronal cells, such as glial cells and endothelial cells, play crucial roles in brain development and function, and their mitochondrial dysfunction may contribute significantly to the pathogenesis of NDDs. For example, glial cells are involved in synaptic support, neurotransmitter metabolism, and immune regulation, and their mitochondrial dysfunction can exacerbate neuronal damage through neuroinflammation and impaired synaptic function. Therefore, future research needs to further explore the specific mechanisms of nonneuronal cell mitochondrial dysfunction in NDDs and its interaction with neuronal dysfunction.

Moreover, developing therapeutic strategies targeting mitochondrial dysfunction is an important direction for future research. Mitochondrial dysfunction in NDDs is often characterized by impaired mitochondrial dynamics, oxidative stress, and defective mitophagy. Several potential therapeutic approaches have been identified, including the use of antioxidants, mitochondrial biogenesis stimulators, and modulators of mitochondrial dynamics. For example, natural polyphenolic compounds such as resveratrol and curcumin have been shown to induce mitochondrial biogenesis and alleviate oxidative stress. Additionally, targeting the PGC-1α pathway, which regulates mitochondrial biogenesis, and the NRF2-antioxidant response element pathway, which enhances cellular antioxidant defenses, has shown promise in preclinical studies. However, translating these findings into effective clinical treatments remains challenging owing to the complexity of mitochondrial dysfunction and the heterogeneity of NDDs.

In summary, while significant progress has been made in understanding the role of mitochondrial dysfunction in NDDs, future research should also focus on exploring the mechanisms in nonneuronal cells and developing targeted therapeutic strategies. A multidisciplinary approach, combining molecular biology, pharmacology, and clinical research, is needed to better understand the underlying pathophysiology and improve outcomes for patients with NDDs.

## Limitations

This review has several limitations. Papers and articles published up to February 20, 2025, and written in English were included, which may have led to publication bias. Other studies or groups that could contribute insights into mitochondrial dynamics dysfunction in NDD may have been excluded from this review. In addition, the conclusions regarding mechanisms drawn from some of the selected studies may originate from animal models rather than humans, such as in the discussion of certain drugs that are still in the exploratory phase. However, these findings still hold significant value in guiding our understanding of the mechanisms involved.

## Conclusion and Perspective

An increasing number of NDDs have been found to be associated with mitochondrial dysfunction. In recent years, new mitochondria-related proteins or other mutations that induce mitochondrial dysfunction have been identified in a variety of NDDs, providing evidence in support of a better understanding of the mechanisms by which mitochondrial dysfunction affects NDD. This paper reviews the role of mitochondrial proteins in mitochondrial bioenergetics, biokinetics, mitochondrial autophagy, and the mitochondrial genome in the pathogenesis of NDD, with a strong emphasis on neurodevelopmental deficits involved in mitochondrial fusion/fission cycle cycling and aberrant autophagic clearance following mitochondrial dysfunction and damage. Thus, improving mitochondrial dysfunction, particularly by modulating the mitochondrial dynamics-related proteome, is a promising therapeutic approach for the treatment of various NDDs.

In addition to describing the mechanisms by which diseases are induced by mitochondrial disorders, we have summarized the molecularly targeted drugs currently used for each mechanism and the diseases they treat. However, many challenges remain. Although an increasing number of pathogenic mechanisms have been identified and are contributing to diagnosis, the precise molecular mechanisms linking mitochondria to the pathogenesis of NDD remain to be elucidated. In addition, mitochondrial mechanisms may differ between different types of NDDs, and further in-depth studies are needed to develop highly specific mitochondrion-targeted NDD therapeutics. To our knowledge, the pathogenesis of neurodevelopmental disorders is still in the exploratory phase. Mitochondrial dysfunction, a pathological change that spans the entire life cycle, has been proven to be highly correlated with NDDs in multiple processes. However, pathological changes in mitochondria often lack disease specificity, and NDD lesions frequently occur early in life. Therefore, clarifying the pathogenesis of NDDs and exploring more disease-specific early indicators are particularly important for the early diagnosis and treatment of NDDs. We anticipate that the discovery of early interventions that can effectively combat mitochondrial dysfunction will promote neuroregeneration and, to the greatest extent possible, alleviate patients’ symptoms and improve adverse prognoses. This is highly important for patients with NDDs, their families, and society as a whole.

Mitochondrial dysfunction plays a significant role in the pathogenesis of neurodevelopmental disorders. By delving into the mechanisms of mitochondrial bioenergetics, biodynamics, autophagy, and the mitochondrial genome, we can lay a theoretical foundation for the development of new therapeutic strategies. Future research needs to further elucidate the precise molecular mechanisms linking mitochondrial dysfunction to neurodevelopmental disorders and explore new therapeutic targets for neural regeneration. Additionally, the pharmacological and technical evaluation of mitochondrial therapies remains challenging. As the field of mitochondrial pharmacology continues to evolve, improving our understanding of mitochondrial dysfunction in neurodevelopmental disorders is crucial for developing new preventive and therapeutic strategies. The treatment strategies for NDDs need to consider the heterogeneity of the diseases and individual differences. Future research should integrate multiomics technologies to analyze the pathological mechanisms of different NDD patients in detail and develop personalized treatment plans. For example, by establishing patient-specific iPSC models, disease progression can be better simulated, and effective therapeutic targets can be screened.

In summary, research on restoring mitochondrial function and promoting neuroregeneration offers new directions for the treatment of NDDs. Through interdisciplinary research and clinical translation, more effective therapeutic approaches are expected to be developed for patients with NDDs.

## Data Availability

*Not applicable*.
